# Dynamic auxin maxima regulate male-to-hermaphrodite conversion and de novo meristem formation in the fern *Ceratopteris* gametophytes

**DOI:** 10.1371/journal.pbio.3003592

**Published:** 2026-01-23

**Authors:** Dinh Nhan Lai, Xi Yang, Chong Xie, Ting Li, An Yan, Xing Liu, Yun Zhou

**Affiliations:** 1 Department of Botany and Plant Pathology, Purdue University, West Lafayette, Indiana, United States of America; 2 Purdue Center for Plant Biology, Purdue University, West Lafayette, Indiana, United States of America; 3 Division of Biology and Biological Engineering, California Institute of Technology, Pasadena, California, United States of America; 4 Howard Hughes Medical Institute, California Institute of Technology, Pasadena, California, United States of America; 5 Department of Biochemistry, Purdue University, West Lafayette, Indiana, United States of America; University of California San Diego, UNITED STATES OF AMERICA

## Abstract

Land plants alternate between generations of asexual sporophytes and sexual gametophytes. Unlike seed plants, ferns produce free-living gametophytes that grow independently from their sporophytes. Gametophytes of the model fern *Ceratopteris* exist in two sex types: hermaphrodites and males. Hermaphrodites maintain meristems and secrete the pheromone antheridiogen, inducing undecided gametophytes to become males. In the absence of antheridiogen, males exhibit developmental plasticity and dynamic cell fate specification by initiating de novo meristems to convert into hermaphrodites. Despite its essential role, the molecular signals governing this process remain unclear. Here, we show that local auxin biosynthesis, dynamically regulated during sex-type conversion, establishes new auxin maxima that are critical for specifying and promoting the proliferation of the meristem progenitor cell (MPC) lineage, ultimately enabling the de novo formation of a multicellular meristem from a single MPC. Time-lapse imaging revealed that upon antheridiogen removal, auxin signaling is specifically activated at the initial site of proliferation in *Ceratopteris* males, triggering new meristem formation. This auxin signaling subsequently becomes concentrated at the center of the proliferating meristem, aligning with localized auxin biosynthesis and the emergence of the meristem notch. Computationally reconstrued lineage maps further showed that chemical inhibition of CrTAA1 abolishes these dynamic auxin patterns, blocking MPC lineage initiation and its subsequent proliferation. Furthermore, genetic knockout of *CrTAA1* via CRISPR-Cas9 phenocopies the effects of chemical inhibition, preventing new meristem formation and disrupting male-to-hermaphrodite conversion. Together, these findings uncover a molecular mechanism underlying sex-type conversion in land plants and highlight the pivotal role of de novo auxin biosynthesis in orchestrating cell fate and proliferation during meristem formation.

## Abstract 2

Ferns alternate between generations of asexual sporophytes and sexual free-living gametophytes. This study uncovers a molecular mechanism underlying male-to-hermaphrodite conversion in a model fern species showing de novo auxin biosynthesis role in orchestrating cell fate and proliferation during meristem formation.

## Introduction

Land plants alternate between asexual sporophyte and sexual gametophyte generations. Unlike seed plants, seed-free plants such as ferns produce gametophytes that are free-living, autotrophic, and develop independently from the sporophytes [1,2]. The model fern *Ceratopteris richardii* (hereafter *Ceratopteris*) exhibits two sex types—male and hermaphrodite—during its haploid gametophyte phase [2–4]. Like many other fern species, Ceratopteris uses the pheromone antheridiogen to regulate sexual differentiation within gametophyte populations [2,3,5]. Ceratopteris hermaphrodites maintain a multicellular meristem, develop egg-producing archegonia, and secret antheridiogen, which induces neighboring sexually undetermined gametophytes to develop as males [2,3,5]. With continuous exposure to antheridiogen, male gametophytes retain their identity, remain ameristic, and differentiate multiple sperm-producing antheridia. Upon maturation, these antheridia release motile sperm that fertilize with eggs produced by hermaphrodites. In contrast, in the absence of antheridiogen, previously determined males gradually lose their male identity and transition into hermaphrodites [3,6,7]. This male-to-hermaphrodite conversion is characterized by the de novo formation of a meristem and subsequent development of egg-bearing archegonia [3,8]. This naturally occurring developmental transition helps maintain a balanced sex ratio within gametophyte populations, promoting outcrossing and ensuring efficient sexual reproduction. A recent study revealed that during sex-type conversion, the entire newly formed meristem originates from a single meristem progenitor cell (MPC) [9]. The MPC lineage sustains high mitotic activity and plays an essential role in promoting this conversion [9]. Despite this understanding, the molecular mechanisms and regulatory factors governing this developmental process remain largely unknown.

The phytohormone auxin, primarily indole-3-acetic acid (IAA), regulates diverse aspects of plant growth and development, spanning both vegetative and reproductive phases [10,11]. Auxin gradients serve as positional cues for organogenesis, including root patterning, phyllotaxis, and leaf venation [12–14]. In flowering plants, morphological changes during sexual differentiation are driven by alterations in auxin levels, mediated by auxin transport, auxin signaling responses, or local auxin biosynthesis [15,16]. The canonical tryptophan (Trp)-dependent indole-3-pyruvic acid (IPA) pathway serves as a key contributor to auxin biosynthesis [17–22]. In this pathway, TRYPTOPHAN AMINOTRANSFERASE OF ARABIDOPSIS 1 (TAA1) and related homologs (TAR1/2) convert Trp to IPA, and YUCCA (YUC) flavin monooxygenase-like proteins catalyze the subsequent rate-limiting step to produce IAA [17–22].

In seed plants, local auxin biosynthesis plays critical roles in meristem initiation, maintenance, and differentiation [23–25]. In *Arabidopsis* shoot apices, TAA1/TAR2-mediated auxin biosynthesis promotes shoot patterning, maintains the shoot meristem size, and regulates stem cell differentiation [25]. Similarly, defects in auxin biosynthesis cause reduced root meristem size and abnormal enlargement of stem cells [23]. Moreover, TAA1-mediated local auxin biosynthesis is required for regenerating root stem cell niches in *Arabidopsis* [24]. In addition to meristem development, auxin biosynthesis also regulates sexual organ development and germ cell formation [26–30]. In seed-free nonvascular plants, genetic studies in bryophyte model species have demonstrated that auxin biosynthesis is crucial for meristem development and body formation during the sexual gametophyte phase [31,32]. For instance, in the liverwort *Marchantia polymorpha*, the *TAA1* homolog, *MpTAA* is specifically expressed in the apical notch meristem [31]. Loss-of-function *Mptaa* gametophytes show severely impaired cell and tissue differentiation, whereas overexpression of *MpTAA* or *MpYUC*2 mimics the effects of exogenous auxin treatment, resulting in growth inhibition, increased rhizoid formation, and disrupted tissue patterning [31]. These findings highlight the essential role of the TAA1-mediated Trp-dependent auxin biosynthesis pathway in *Marchantia* gametophyte development [31]. Consistently, in the moss *Physcomitrella patens*, shoot apical cells serve as the center for auxin biosynthesis, which regulates subsequent cell growth and egg cell differentiation in descendant cells [32]. Together, these genetic studies suggest conserved roles of localized auxin biosynthesis in meristem development and tissue differentiation across bryophyte gametophytes [31–33].

In ferns, which represent seed-free vascular plants [34,35], auxin has been detected in developing gametophyte bodies of *Polystichum aculeatum* [36]. Drug treatments and exogenous auxin applications further suggest important roles for auxin during the sexual gametophyte phase in ferns [36–39]. For instance, exogenous auxin promoted protonema growth but inhibits antheridium formation in *Lygodium japonicum* gametophytes [37], and negatively affected general prothallus growth, gametophyte morphology, and wound regeneration in Ceratopteris hermaphrodites [38,39]. These findings imply that the auxin responses in fern gametophytes share key features with those observed in bryophyte gametophytes [31,38], indicating that precisely regulated auxin levels likely play essential roles in maintaining Ceratopteris gametophyte development. However, spatial-temporal dynamics of auxin production and signaling in Ceratopteris gametophytes remain largely unknown, and genetic analyses of auxin function during the gametophyte phase are also lacking.

In this study, we combined time-lapse confocal imaging, computational analysis, chemical perturbation, and CRISPR-Cas9 gene-editing in transgenic Ceratopteris to demonstrate that de novo auxin biosynthesis is essential for male-to-hermaphrodite conversion in Ceratopteris gametophytes. Removal of antheridiogen rapidly and locally activated auxin biosynthesis, establishing a new auxin-signaling maximum that marked an early molecular signature of sex-type conversion. This auxin maximum was appeared at the initiation sites of the future hermaphrodite and later became confined to the meristem notch, where drove cell-cycle re-entry and sustained proliferation, ultimately generating a multicellular meristem from a single MPC. Chemical inhibition of CrTAA1 activity, or genetic knockout of *CrTAA1* via CRISPR-Cas9, disrupted MPC lineage establishment and de novo meristem formation, thereby significantly reducing sex-type conversion. These findings reveal an evolutionarily conserved auxin biosynthesis pathway crucial for sex expression and meristem formation in haploid gametophytes, highlighting both conserved and lineage-specific mechanisms regulating cell division and fate specification in land plants.

## Results

### Auxin signal dynamics during Ceratopteris hermaphrodite and male development and male-to-hermaphrodite conversion

To visualize auxin signals in Ceratopteris gametophytes, we used the DR5 synthetic promoter, which contains tandem repeats of auxin response elements that marks active auxin signaling sites [40]. Specifically, we stably transformed Ceratopteris with the well-established *DR5v2::ntdTomato* reporter construct [41] and isolated multiple independent transgenic lines that exhibited comparable expression patterns in Ceratopteris gametophytes. To test whether the DR5 reporter was responsive to auxin in Ceratopteris gametophytes, we performed live-imaging experiments with IAA treatment. At 3 days after germination (DAG), individual males and hermaphrodites from the *DR5v2::ntdTomato* reporter line were transferred to medium supplemented with either mock treatment or 10 µM IAA (S1A–S1P Fig). After 24 h of IAA treatment, we found that, in contrast to the localized DR5 expression patterns observed in mock-treated controls (S1A–S1H Fig), DR5 signals became broadly distributed across nearly all cell in both male and hermaphrodite gametophytes (S1I–S1P Fig). Heat maps clearly illustrated significantly elevated DR5 signals in response to exogenous IAA compared to mock controls (S1A–S1D and S1I–S1L Fig). These results confirm that the DR5 reporter is functional and responsive to auxin signaling in Ceratopteris gametophytes.

**Fig 1:**
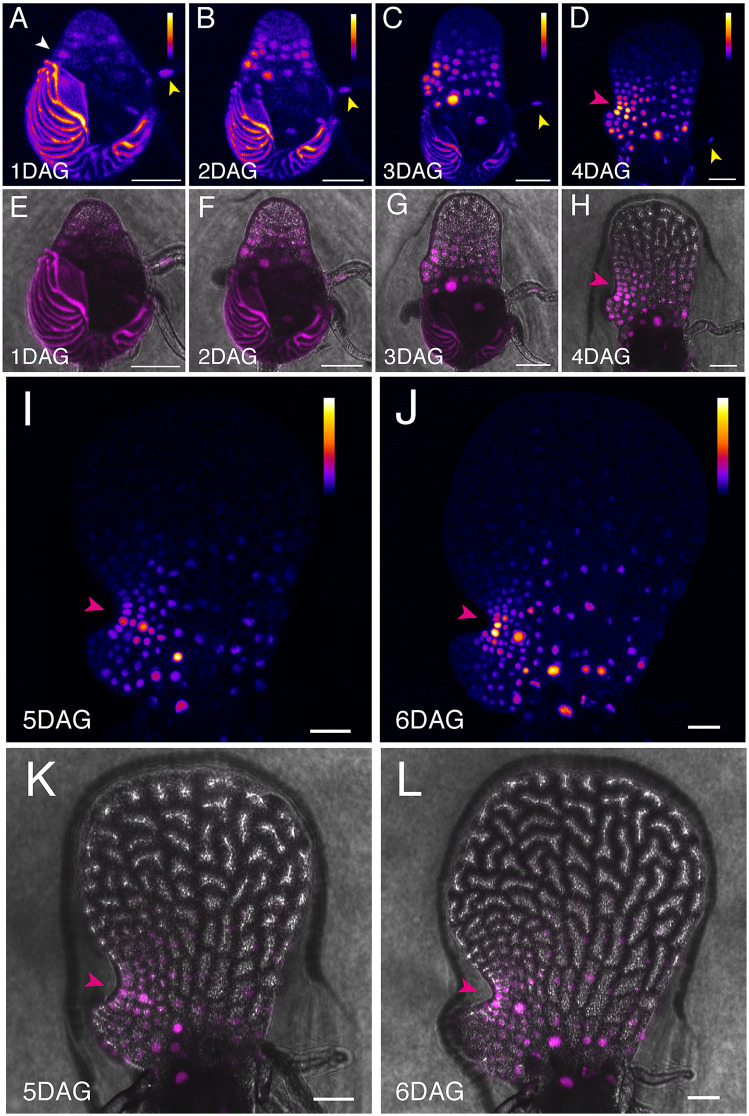
Auxin signaling dynamics during Ceratopteris hermaphrodite development. **(A–L)** Confocal time-lapse images showing auxin signaling dynamics in Ceratopteris hermaphrodite gametophytes expressing the *DR5v2::ntdTomato* transgenic reporter. A hermaphrodite (A–L) was live-imaged every day from 1 to 6 days after germination (DAG). (A–D, I–J) Z-projection views of tdTomato signals (Fire LUT). (E–H, K–L) Merged channels of tdTomato (magenta) and DIC (gray, showing cell outlines). The white arrowhead (A) indicates the initial DR5 signals in the early developing prothallus, while yellow arrowheads (A–D) indicate DR5 signals in developing rhizoids. Magenta arrowheads (D, H, I–L) highlight the auxin signaling peak during the initiation and establishment of a multicellular meristem in the hermaphrodite. Color bars (A–D, I–J) represent the relative intensity of DR5 signals in Fire LUT images, with low intensity shown in black, intermediate intensity in blue to red, and high intensity in yellow to white. The color scale applies to the entire prothallus but excludes the spore coat. Scale bars: 50 µm. Three independent samples were analyzed, showing comparable results.

**Fig 2:**
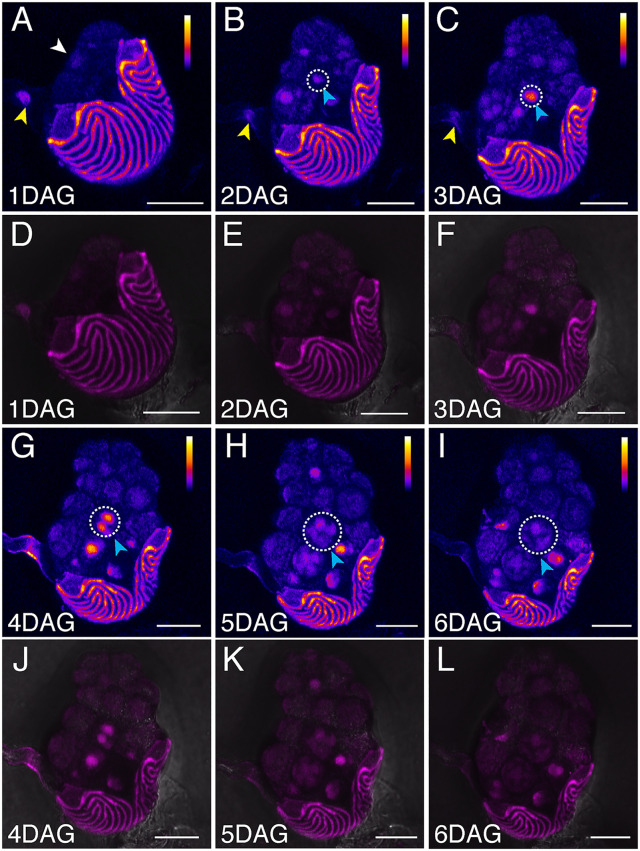
Auxin signaling dynamics during Ceratopteris male development in response to antheridiogen. **(A–L)** Confocal time-lapse images showing auxin signaling dynamics in Ceratopteris male gametophytes expressing the *DR5v2::ntdTomato* transgenic reporter, grown on CFM. A male gametophyte was live-imaged every day from 1 to 6 DAG. (A–C, G–I) Z-projection views of tdTomato signals (Fire LUT). (D–F, J–L) Merged channels of tdTomato (magenta) and DIC (gray, showing cell outlines). The white arrowhead (A) indicates the initial DR5 signal in the early developing prothallus, while yellow arrowheads (A–C) indicate DR5 signals in developing rhizoids. Cyan arrowheads and white dashed circles (B–C, G–I) highlight DR5 signaling dynamics during antheridium initiation and maturation. Color bars (A–C, G–I) represent the relative intensity of DR5 signals in Fire LUT images, with low intensity shown in black, intermediate intensity in blue to red, and high intensity in yellow to white. The color scale applies to the entire prothallus but excludes the spore coat. Scale bars: 50 µm. Three independent samples were analyzed, showing comparable results.

We then performed non-invasive, time-lapse imaging to analyze auxin signaling dynamics during Ceratopteris hermaphrodite and male gametophyte development. Spores of the *DR5v2::ntdTomato* transgenic reporter line were plated on fern medium (FM) or conditioned FM (CFM, containing antheridiogen) to observe hermaphrodite and male development, respectively. Once germinated, hermaphrodite samples were live-imaged every day on the original FM plate from 1 to 6 DAG. At 1 DAG, DR5 signals were first detected in the developing prothallus (white arrowhead, Fig 1A), as well as in the rhizoid (yellow arrowhead). By 2 DAG, the DR5 reporter expression was localized to the basal part of the hermaphrodite (Fig 1B). From 3 to 6 DAG, corresponding to meristem initiation and proliferation, DR5 expression gradually formed an intensity gradient, with peak expression at the center of the notch meristem and lower expression further away from the notch (Magenta arrowheads, Fig 1C, 1D, and 1G–1L). DR5 signals were also observed in developing archegonia (Fig 1I–1L). In contrast, in males germinated and grown on CFM, DR5 signals were generally weaker compared to hermaphrodites on FM, suggesting that antheridiogen, the male differentiation inducer, negatively influences auxin signaling in males (Fig 2A–2L). Males and hermaphrodites exhibited differences in auxin signal distribution beginning at 2 DAG (Figs 1B, 1F, 2B, and 2E). We observed new auxin signals in the cells that eventually differentiated into the antheridium (cyan arrowheads, Fig 2). As the antheridia matured, DR5 signal intensity decreased, exhibiting a temporary increase followed by a reduction in signal intensity in the differentiating antheridium cells (Fig 2B, 2C, and 2G–2I). These dynamic expression patterns, especially the DR5 signal peak at the center of actively proliferating multicellular meristems, suggest potential roles of auxin in regulating Ceratopteris gametophyte development.

**Fig 3:**
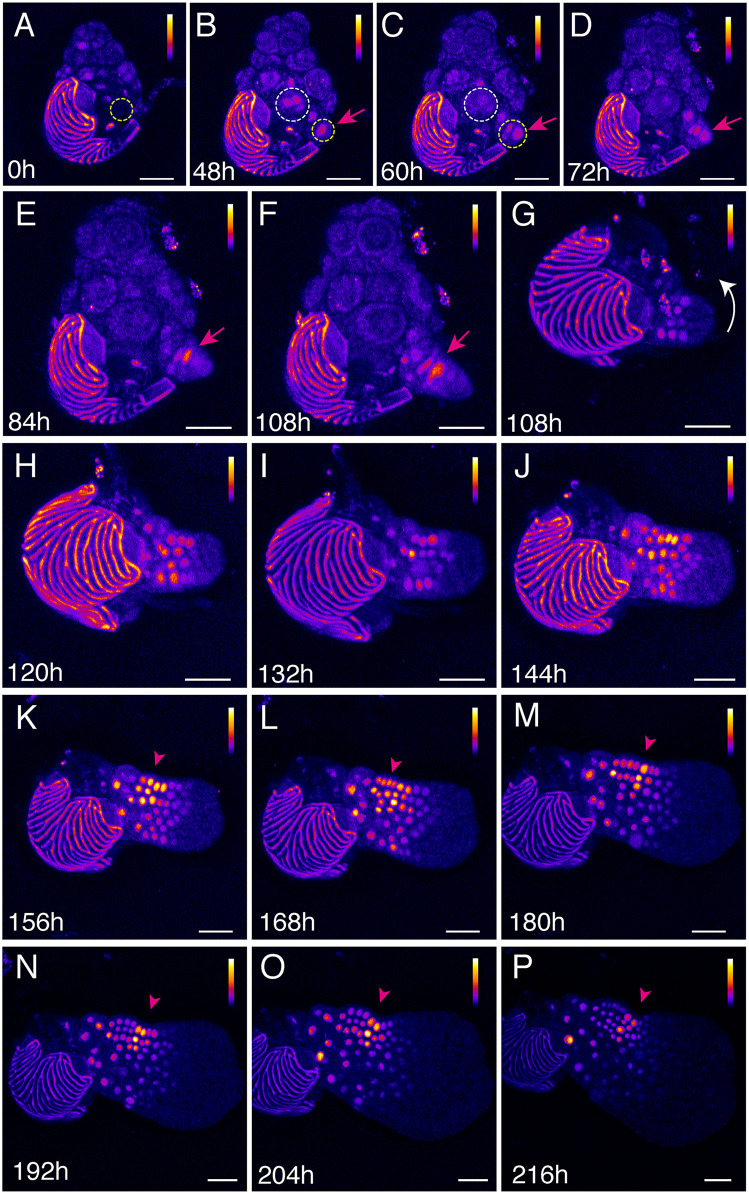
Auxin signaling dynamics during male-to-hermaphrodite conversion and de novo meristem formation in the absence of antheridiogen. **(A–P)** Confocal time-lapse images of a Ceratopteris gametophyte expressing the *DR5v2::ntdTomato* reporter. A 3-DAG male gametophyte was first imaged at 0 h (0 h) after being transferred to an antheridiogen-free medium and subsequently live-imaged at multiple time points from 48 to 216 h after the transfer. (A–P) Z-projection views of tdTomato signals (Fire LUT). White dashed circles (B–C) mark regions with reduced DR5 signals corresponding to antheridium differentiation. Yellow dashed circles (A–C) highlight the region with newly merged auxin signals at the site of de novo hermaphrodite formation. Magenta arrows (B–F) indicate the emergence (B) and persistence (C–F) of DR5 signals at the initiation site of the newly forming hermaphrodite. Magenta arrowheads (K–P) indicate dynamic DR5 signal patterns associated with de novo multicellular meristem formation. A white curved arrow (G) indicates the adjusted orientation of the sample at the same point (108 h, G) for clear visualization of the newly formed hermaphrodite body. Color bars (A–P) represent the relative intensity of DR5 signals in Fire LUT images, with low intensity shown in black, intermediate intensity in blue to red, and high intensity in yellow to white. The color scale applies to the entire prothallus but excludes the spore coat. Scale bars: 50 µm. Three independent samples were analyzed, showing comparable results.

Next, we performed time-lapse imaging to investigate the DR5 reporter expression patterns during male-to-hermaphrodite conversion (Fig 3A–3P), comparing them with patterns observed during normal male and hermaphrodite development (Figs 1A–1L and 2A–2L). *DR5v2::ntdTomato* spores were surface-sterilized and plated on CFM to induce male differentiation, as described in previous studies [8,9,42]. Individual male gametophytes at 3 DAG were transferred to antheridiogen-free FM, with one gametophyte per plate to initiate de novo meristem formation without potential interference from antheridiogen released by neighboring hermaphrodites. Confocal live-imaging was performed immediately after transfer (0 h after transfer, 0 h; Fig 3A) and continued at multiple time points from 48 to 216 h after transfer, capturing the initiation and establishment of new hermaphrodites (Fig 3B–3P). At 0 h, weak DR5 signals were observed in developing antheridia (Fig 3A), similar to those observed in males continuously grown on CFM (Fig 2B, 2C, 2E, and 2F). These signals gradually diminished as each antheridium matured (Fig 3B–3F; white dashed circles in Fig 3B and 3C). At subsequent time points, no new antheridium-differentiated cells appeared, and no further DR5 signals were detected within existing antheridia (Fig 3B–3F), consistent with the previous observation that continuous exposure to antheridiogen is necessary for new antheridium initiation [5]. More importantly, time-lapse imaging revealed that the emergence of new DR5 signals at 48 h after transfer (yellow circles, Fig 3A and 3B), which persisted (yellow circle, Fig 3C) and continued to expand, correlating with the initiation and growth of the new hermaphrodite body (magenta arrowheads, Fig 3B–3F). By 108 h, DR5 signals were highly expressed at the basal region of the newly formed hermaphrodite (Magenta arrows, Fig 3F and 3G), mimicking to expression patterns observed during normal hermaphrodite development (Fig 1B and 1C). DR5 signals maintained highly expressed in the base region of the newly formed hermaphrodite throughout its expansion and gradually formed a distinct intensity gradient as the de novo meristem was initiated and became established (Fig 3H–3P). Heat maps revealed that, once a concave meristem notch formed, DR5 signals peaked at the center of the notch (arrowheads, Fig 3K–3P). In summary, auxin signaling dynamics are directly associated with the initiation of male-to-hermaphrodite conversion (Fig 3B–3F) and the subsequent meristem initiation and notch formation (Fig 3K–3P).

### Auxin biosynthesis is essential for male-to-hermaphrodite conversion

**Fig 4:**
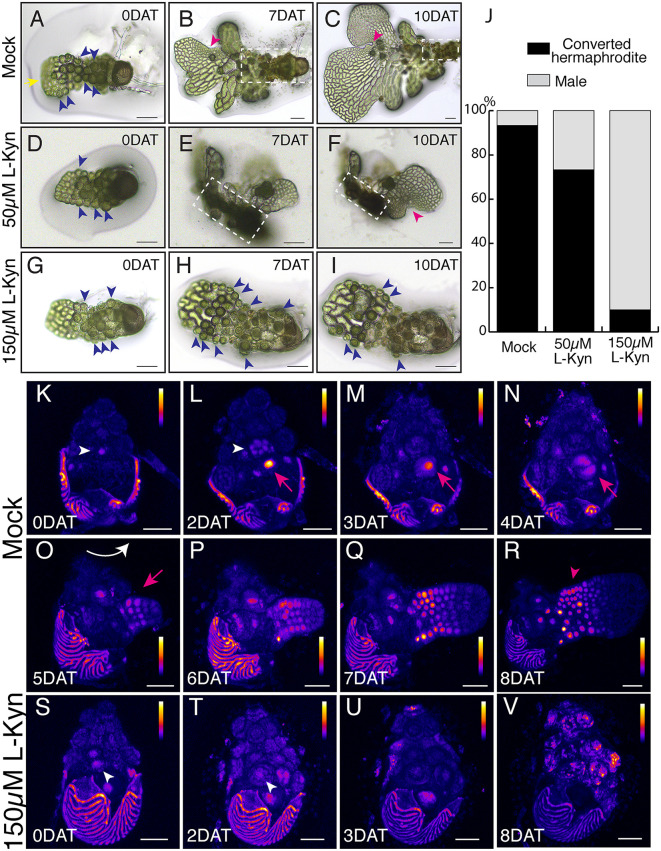
Auxin biosynthesis inhibition blocks male-to-hermaphrodite conversion in the absence of antheridiogen. Light micrographs of representative male gametophytes at 0, 7, and 10 days after transfer (DAT) to antheridiogen-free FM, treated with either mock **(A–C)**, 50 µM L-kynurenine **(D–F)**, or 150 µM L-kynurenine (G–I). In the mock-treated sample, the yellow arrow (A) indicates the site where a meristem and a hermaphrodite body begin to form de novo. White dashed rectangles (B–C) indicate the original male body observed in **(A)**. Blue arrowheads (A, D, G–I) indicate several antheridia, while magenta arrowheads (B–C, F) indicate the newly formed meristem. **(J)** The percentage of the successfully converted hermaphrodites out of the total samples examined was calculated from three groups of experiments, each containing 20 independent gametophytes (60 in total). The data underlying panel (J) can be found in S1 Data. (K–V) Confocal time-lapse images of Ceratopteris gametophytes expressing the *DR5v2::ntdTomato* reporter. 3-DAG male gametophytes were first imaged at 0 DAT to antheridiogen-free FM **(K, S)**, treated with either mock (K) or 150 µM L-kynurenine **(S)**, and then live-imaged at multiple time points from 2 to 8 DAT (K–R, S–V). (K–V) Z-projection views of tdTomato signals (Fire LUT). White arrowheads (K–L, S–T) indicate DR5 signals in the antheridia of male gametophytes. Magenta arrows indicate the site of the de novo meristem and hermaphrodite body formation in the mock-treated sample **(M–O)**. The magenta arrowhead (R) indicates DR5 signals in the newly formed multicellular meristem. The white curved arrow (O) indicates the adjusted orientation of the mock-treated sample from 4 DAT to 5 DAT for clear visualization of the developing hermaphrodite. Color bars (K–V) represent the relative intensity of DR5 signals in Fire LUT images (K–V), with low intensity shown in black, intermediate intensity in blue to red, and high intensity in yellow to white. The color scale applies to the entire prothallus but excludes the spore coat. Scale bars: 100 µm (A–I) and 50 µm (K–V). Three independent samples from each treatment were analyzed, showing comparable results.

To explore the role of auxin, particularly the establishment of new peak of auxin signaling, in controlling male-to-hermaphrodite conversion and associated de novo meristem formation, we first employed chemical perturbation on new auxin production. L-Kynurenine (Kyn) is a specific inhibitor of TAA1-mediated auxin biosynthesis, as previously demonstrated in the flowering plant model *Arabidopsis* and the seed-free plant model *Marchantia* [31,43]. Kyn therefore served as a valuable tool to assess how disrupted auxin biosynthesis affects sex-type conversion and meristem initiation in Ceratopteris gametophytes. For this test, individual 3-day-old WT male gametophytes initially germinated on CFM were transferred onto antheridiogen-free FM plates (one male per plate) supplemented with either mock or Kyn at concentrations of 50 or 150 µM (Fig 4A–4I). This experimental design allowed us to directly evaluate the effects of Kyn treatment on male-to-hermaphrodite conversion relative to mock controls (Fig 4A–4I). Light microscopy images of these samples were captured immediately after transfer (0 days after transfer [DAT]) and subsequently at 7 and 10 DAT. Our results demonstrated a clear, dosage-dependent inhibitory effect of Kyn on male-to-hermaphrodite conversion (Fig 4J and S1 Data). Mock-treated controls exhibited no inhibition, achieving over 93% successful conversions of sex types (Fig 4A–4C, 4J, and S1 Data). In contrast, treatment with 50 µM Kyn reduced the conversion rate to approximately 73% (Fig 4J and S1 Data), and the resulting hermaphrodites were notably smaller compared to mock-treated controls (Fig 4C and 4F). At the higher concentration (150 µM), Kyn strongly inhibited conversion and prevented new meristem formation (Fig 4G–4I), with only 10% of males successfully transitioning to hermaphrodites (Fig 4J and S1 Data). Notably, at this concentration, no meristem structures were observed at 7 DAT, and most males exhibited minimal growth between 7 DAT and 10 DAT (Fig 4H–4I).

We then investigated how Kyn treatment affects DR5 expression patterns following the transfer of males to antheridiogen-free medium. 3-DAG *DR5v2::ntdTomato* male gametophytes were transferred to antheridiogen-free FM plates supplemented with either mock or 150 µM Kyn. In mock-treated controls (Fig 4K–4R), DR5 expression patterns closely resembled those during male-to-hermaphrodite conversion described above (Fig 3), with initial new DR5 peaks overlapping with the initiation site of newly formed hermaphrodites, followed by a clear intensity gradient established in the de novo meristem during sex-type conversion (Fig 4L–4R). In contrast, Kyn treatment substantially diminished auxin signaling dynamics (Fig 4S–4V). Despite transfer to an antheridiogen-free environment, no new proliferation sites or associated DR5 signal were observed in Kyn-treated samples (Fig 4S–4V). These results suggest that reducing auxin biosynthesis by Kyn-mediated inhibition of CrTAA1 diminishes auxin signaling essential for male-to-hermaphrodite conversion.

### Auxin biosynthesis is required for MPC establishment and cell proliferation during de novo meristem formation

**Fig 5:**
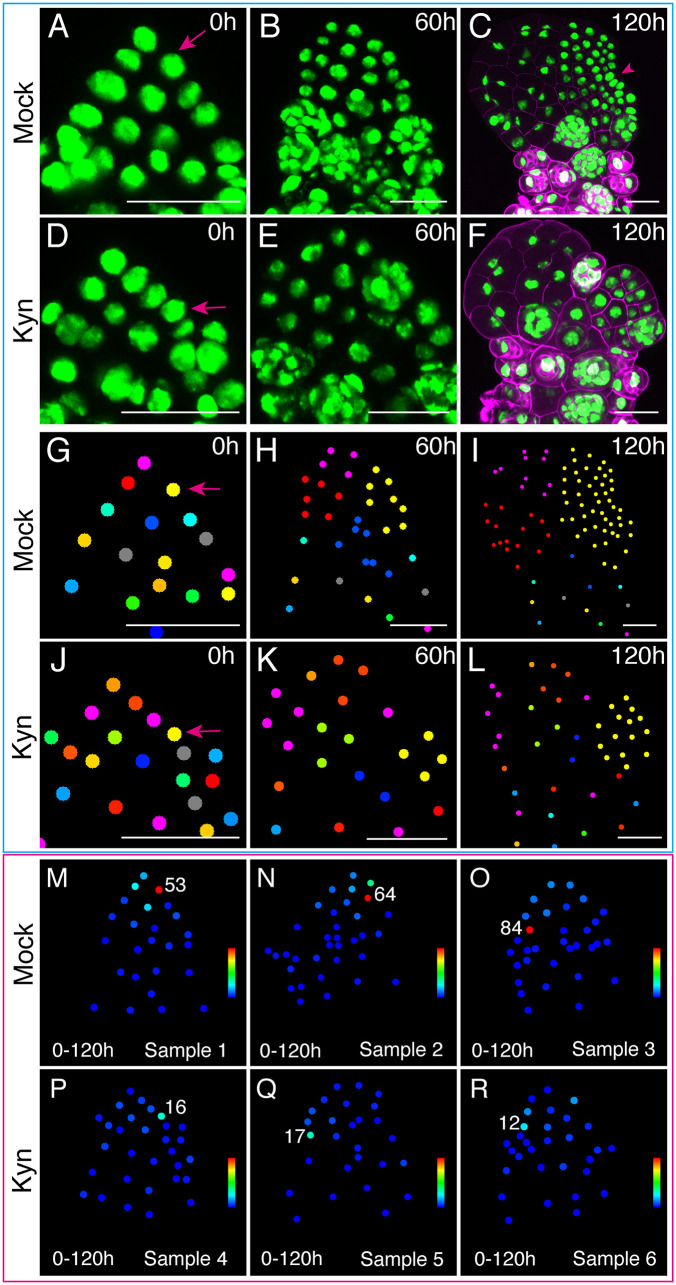
L-kynurenine inhibits the formation of MPC during male-to-hermaphrodite conversion. **(A–F)** Confocal images of 2-DAG Ceratopteris male gametophytes expressing *CrUBQ10p::H2B-GFP::3′CrUBQ10*, taken at 0 (A, D), 60 (B, E), and 120 (C, F) hours after treatment with either mock (sample 1, A–C) or 150 µM Kyn (sample 4, D–F). Green: GFP. (C, F) Merged channels of GFP (green) and propidium iodide (PI) (magenta); PI staining was performed at 120 h to visualize cell outlines. Time-lapse imaging was performed every 6 h, and all time points from 0 to 120 h for sample 1 and sample 4 are shown in S2A–S2V and S5A–S5V Figs, respectively. Panels (A–F) correspond to zoomed-in regions from the supplementary figures: (A) from S2A Fig, (B) from S2K Fig, (C) from S2V Fig; (D) from S5A Fig, (E) from S5K Fig, and (F) from S5V Fig. Three independent biological replicates per treatment were imaged under the same conditions, yielding comparable results for each treatment. Time-lapse imaging results from the other samples are shown in S3 and S4 Figs (mock-treated samples 2 and 3) and S6 and S7 Figs (Kyn-treated samples 5 and 6). **(G–L)** Cell lineage dynamics of live-imaged sample 1 (G–I) and sample 4 (J–L) at 0, 60, and 120 h, corresponding to panels (A–F). Each solid circle represents either a single nucleus or a group of nuclei within one antheridium. At 0 h, adjacent nuclei were labeled with different colors as a reference for visualizing distinct lineages at subsequent time points. Cells and their progeny were labeled with the same color across all time points to represent the same lineage. When an antheridium developed into a 3D complex structure, all nuclei within the same antheridium were represented as a single solid circle to simplify visualization. Magenta arrows highlight either the MPC (yellow) lineage (A, G) contributing to de novo meristem formation in mock-treated sample 1 or the lineage (yellow) (D, J) with the most division events in Kyn-treated sample 4. Full lineage dynamics from 0 to 120 h for samples 1 and 4 are shown in S8A–S8U and S11A–S11U Figs, respectively. Panels (G–I) correspond to zoomed-in regions from the supplementary figures: (G) from S8A Fig, (H) from S8K Fig, and (I) from S8U Fig; (J) from S11A Fig, (K) from S11K Fig, and (L) from S11U Fig. Lineages from three independent samples per treatment were analyzed, showing comparable results for each treatment. Lineage dynamics of the other samples are shown in S9 and S10 Figs (mock-treated samples 2 and 3) and S12 and S13 Figs (Kyn-treated samples 5 and 6). **(M–R)** Quantification of total division events for each cell lineage across all analyzed samples (from S2 to S7 Figs). Each solid circle represents an individual nucleus at 0 h. Numbers indicate the total division events over 120 h within the MPC lineages from mock-treated samples 1-3 (M–O) and the lineages with the most division events from Kyn-treated samples 4-6 (P–R). Total division events are color-coded, ranging from blue (minimum, 0) to red (maximum, > 53). Scale bars (A–R): 50 µm.

Our previous studies demonstrated that upon removal from antheridiogen, male gametophytes transition into hermaphrodites, establishing a newly formed and actively proliferating meristem. This multicellular meristem originates from a single MPC in the original male [9]. To visualize cellular proliferation dynamics at single-cell resolution under conditions where auxin biosynthesis was disrupted, we used the transgenic reporter line *pCrUBQ10::H2B-GFP::3′CrUBQ10*, which ubiquitously expresses a nuclear-localized GFP in all cells, enabling dynamic analysis of individual cells over time [9]. Spores from the reporter line were surface-sterilized and germinated on CFM to induce male differentiation. Two DAG, male gametophytes were transferred onto antheridiogen-free FM supplemented with either mock treatment or with 150 µM of the auxin biosynthesis inhibitor, Kyn (Figs 5A–5F and S2–S7).

Confocal live imaging was performed directly on growing Petri dishes every 6 h, beginning immediately after transfer and continuing for a total of 120 h (Figs 5A–5F and S2–S7). Between imaging intervals, samples were returned to growth chambers and maintained under standard growth conditions (29 °C, continuous light). Consistent with previous observations in WT [9], non-antheridium cells in mock-treated samples actively proliferated after transfer to antheridiogen-free medium, successfully transitioning from males to hermaphrodites by 120 h (Figs 5A–5C and S2–S4). This transition was characterized by the formation of a clear, notch-shaped de novo meristem (Figs 5C, S2U, S2V, S3U, S3V, and S4U). In contrast, Kyn-treated samples, though placed on antheridiogen-free medium, exhibited low proliferation activity and remained male throughout the entire 120-h imaging period (Figs 5D–5F and S5–S7), consistent with results from light microscopy (Fig 4G–4J). These findings demonstrate that Kyn treatment efficiently inhibits the male-to-hermaphrodite transition triggered by antheridiogen-free conditions.

We then applied the previously established pipeline [9,44] to performed 2D imaging analyses on samples treated with either mock or Kyn (Figs 5G–5L and S8–S13). We segmented each GFP-tagged nucleus at various time points and assigned unique IDs to each individual nucleus. This enabled tracing of each nucleus and its descendants over time, generating dynamic lineage maps of all segmented nuclei from 0 to 120 h, corresponding to the period of initiation and formation of the new multicellular meristems in mock-treated samples (Figs 5G–5L, S8, and S10). At 0 h, each nucleus represented an independent lineage, with their clonal descendants sharing with the same color in all subsequent time points (Figs 5G–5L, S8, and S10). Consistent with previous observations in WT samples [9], in mock-treated samples, a single progenitor cell at 0 h, identified as the MPC (magenta arrows, Figs 5G and S8A), gave rise to the entire de novo meristem (Figs 5G–5I and S8A–S8U). The MPC lineage (yellow) progressively expanded, becoming the dominant cell sector (Figs 5G–5I and S8A–S8U) that eventually contributing to all cells within the de novo meristem (Figs 5I, S2U, S2V, and S8U). The MPC lineage represents the most actively dividing cell population, surpassing all other lineages. Conversely, in the Kyn-treated sample, the actively dividing lineages (such as the one in yellow, Figs 5J–5L and S11A–S11U) exhibited a significantly reduced number of progenies, with a much smaller sector compared to the MPC lineage in the mock control at the same time points (Figs 5G–5I and S8A–S8U). Due to the failure in de novo meristem establishment under Kyn treatment, no MPC lineage could be defined (Figs 5J–5L and S11A–S11U). These results were consistent across multiple samples and confirmed through lineage analysis (mock-treated samples 1–3 in S8–S10 Figs versus Kyn-treated samples 4–6 in S11–S13 Figs).

Aligning with lineage maps (Figs 5G–5L and S8–S13), quantitative analyses demonstrated that, over the 120-h period, the total number of division events in MPC lineages from mock-treated samples 1–3 (Fig 5M–5O) consistently exceeded those observed in any lineage from three Kyn-treated samples 4–6, including the most actively dividing lineage (Fig 5P–5R). These results suggest that de novo auxin biosynthesis is crucial for meristem cell proliferation. Detailed 12-h division maps (S14–S18 Figs) further illustrated the inhibitory effects of Kyn treatment on cell proliferation during the male-to-hermaphrodite switch and associated de novo meristem formation, providing high spatial and temporal resolution. In these maps, segmented cells undergoing division within each 12-h interval were highlighted in magenta, while those remaining undivided were shown in green (S14–S18 Figs). Cell division occurring within mature antheridia was not included in this analysis. Specifically, during the first 60 h after transfer (S14–S18 Figs), cell division events were randomly distributed across the apical region in both mock- and Kyn-treated samples, and the MPC lineage in the mock-treated samples (S14A–S14D, S14I, S15A–S15E, and S16A–S16E Figs) did not yet exhibit a clear difference in division activity, which is consistent with the previously defined Phase I of the WT male-to-hermaphrodite conversion [9]. During this phase, Kyn-treated samples showed fewer overall dividing cells (S14E–S14H, S14L, S17A–S17E, and S18A–S18E Figs) compared to mock samples (S14A–S14D, S14I, S15A–S15E, and S16A–S16E Figs), suggesting that auxin may facilitate cells re-entering the cell cycle. During the subsequent 60 h, corresponding to Phase II as previously defined [9], division events in mock-treated samples became gradually localized and eventually restricted to the MPC lineages (S14J, S14K, S14O–S14Q, S15F–S15J, and S16F–S16J Figs). In contrast, the number and pattern of division events in Kyn-treated samples remained unchanged compared to Phase I (S14M, S14N, S14R–S14T, S17F–S17J, and S18F–S18J Figs), and no actively proliferating MPC lineages could be identified. For direct comparison, the most actively dividing non-antheridium cell lineages from each Kyn-treated sample were outlined (white dashed outlines, S14, S17, and S18 Figs), clearly showing fewer divisions compared to the MPC lineage (yellow dashed outlines, S14–S16 Figs) in mock-treated samples. In summary, disrupting auxin biosynthesis efficiently blocks the male-to-hermaphrodite transition by suppressing the initiation and subsequent proliferation of the MPC lineage.

### Dynamic expression patterns of *CrTAA1* during male-to-hermaphrodite conversion

We next set out to identify and characterize TAA1 homologs in *Ceratopteris*. Protein sequences of *Arabidopsis* AtTAA1 and AtTAR homologs were used as references for BLASTp searches against the genomes of *Ceratopteris richardii*, *Azolla filiculoides*, and *Salvinia cucullata*. Previously identified TAA1 and TAR homologs from several representative land plant species [45] were obtained from the Phytozome database. Multiple sequence alignments were performed, and a maximum-likelihood phylogenetic tree was constructed (S19 Fig). Candidate Ceratopteris orthologs were further analyzed with InterProScan and compared to *Arabidopsis* TAA1 family members (S2 Data). Our phylogenetic analysis revealed that only one *C. richardii* gene, *CrTAA1* (Ceric.10G039800), grouped within the AtTAA1/AtTAR1/AtTAR2 lineage (S19 Fig and S2 and S3 Data). Furthermore, *CrTAA1* is the only Ceratopteris gene classified within the *TAA* clade, which is characterized by the exclusive presence of an Alliinase-C domain (IPR006948) (S2 Data). In contrast, all other candidate Ceratopteris orthologs fell within a different (AtTAR3/AtTAR4) clade and possessed both an N-terminal Alliinase-EGF (Epidermal Growth Factor, IPR006947) domain and the Alliinase-C domain (S2 Data). Together, these results demonstrate that *CrTAA1* (Ceric.10G039800) is the sole *TAA1* homolog in the *Ceratopteris* genome.

**Fig 6:**
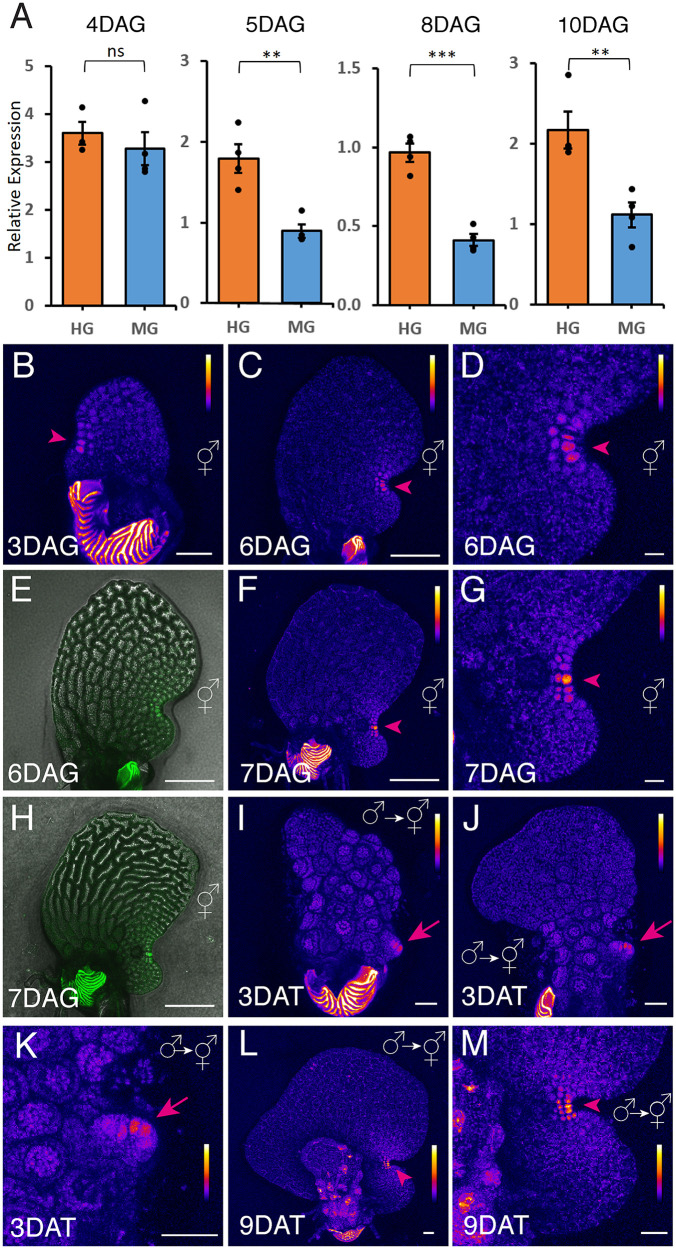
*CrTAA1* expression patterns in Ceratopteris gametophytes. **(A)** Quantitative PCR analysis showing the relative expression levels of *CrTAA1* (normalized to the internal control *CrACTIN1*) in hermaphroditic gametophytes (HG) and male gametophytes (MG) at the indicated DAG. Bar: mean ± sem (*n* = 4 biological replicates for 4-DAG MG, 5-DAG HG or MG, 8-DAG HG or MG, and 10-DAG HG or MG; *n* = 3 biological replicates for 4-DAG HG). ns, not significant (*p* > 0.05); ** *p* < 0.01; *** *p* < 0.001. Each biological replicate included pooled hermaphrodites or males collected at the indicated DAG. The data underlying panel (A) can be found in S4 Data. **(B–H)** Confocal images of *pCrTAA1::H2B-GFP::3′CrTAA1* reporter expression in different hermaphrodites at the indicated DAG. (C–E) One hermaphrodite at 6 DAG. (F–H) One hermaphrodite at 7 DAG. (D, G) Zoomed-in views of the meristems of the hermaphrodites shown in (C) and (F), respectively. (I–M) Confocal images of different gametophytes during male-to-hermaphrodite conversion, expressing the *pCrTAA1::H2B-GFP::3′CrTAA1* reporter, taken at indicated days after transfer (DAT) to antheridiogen-free medium. (I, J) Gametophytes at 3 DAT. (K) Zoomed-in view of the initiation site of hermaphrodite formation shown in (J). (L, M) A newly formed hermaphrodite at 9 DAT. (M) Zoomed-in view of the de novo meristem shown in (L). Magenta arrowheads (B–D, F–G, L–M) indicate *CrTAA1* expression specifically in the notch region of multicellular meristems during normal hermaphrodite development (B–D, F–G) and in de novo formed meristems during male-to-hermaphrodite conversion (L–M). Magenta arrows (J, K) indicate GFP signals at the initiation site of de novo meristem formation. (B–D, F–G, I–M) Z-projection views of GFP signals (Fire LUT). **(E, H)** Merged channels of GFP (green) and DIC (gray, showing cell outlines). Scale bars: 50 µm. Color bars (B–D, F–G, I–M) represent the relative intensity of GFP signals in Fire LUT images, with low intensity shown in black, intermediate intensity in blue to red, and high intensity in yellow to white. The color scale applies to the entire prothallus but excludes the spore coat.

We initially characterized the expression dynamics of *CrTAA1* using quantitative PCR (qPCR) (Fig 6A and S4 Data). Wild-type (WT) spores were plated on FM, and RNA was separately extracted from male and hermaphroditic gametophytes harvested from the same Petri dishes at 4, 5, 8, or 10 DAG. This allowed for a direct comparison of *CrTAA1* expression levels in males and hermaphrodites over time. At each time point, males or hermaphrodites were pooled as a single biological replicate, with at least three biological replicates included to assess *CrTAA1* expression at each stage. *CrACTIN1* was used as the reference gene, as described previously [42]. *CrTAA1* transcripts were consistently detected at all time points, with expression observed at 4, 5, 8, and 10 DAG during the gametophyte stage (Fig 6A and S4 Data). Notably, starting at 5 DAG, when meristems are established in hermaphrodites, *CrTAA1* expression was significantly higher in hermaphrodites compared to males at the same DAG. This suggests that *CrTAA1* expression correlates to meristem development in hermaphrodites.

Next, we generated a transgenic fluorescent transcriptional reporter for *CrTAA1* (*pCrTAA1::H2B-GFP::3′CrTAA1*) to investigate its expression dynamics with higher spatial resolution (Fig 6B–6M). First, we focused specifically on *CrTAA1* expression patterns during hermaphrodite development, performing confocal live imaging of hermaphrodite samples at various DAG (Fig 6B–6H). Quantitative imaging results revealed that the *CrTAA1* reporter exhibited relatively weak, yet highly specific, expression patterns (Fig 6B–6H). At 3 DAG, *CrTAA1* expression localized specifically to one side of the hermaphrodite, overlapping with the initiation site of the multicellular meristem (Fig 6B). As the meristem further developed, *CrTAA1* expression became exclusively confined to the central region of the meristem, particularly concentrated at the meristem notch region (Fig 6C-6H). Heat maps showed an expression gradient, with peak *CrTAA1* expression at the notch center (Fig 6D and 6G). These findings suggest that during meristem establishment and notch formation in hermaphrodites, auxin biosynthesis is locally upregulated at the meristem center through at least the TAA1 pathway. Then, we examined *CrTAA1* reporter expression during the male-to-hermaphrodite transition and de novo meristem formation. Males of the *CrTAA1* reporter line, initially germinated on CFM, were transferred onto antheridiogen-free FM and imaged at different DAT. By 3 DAT, when males began initiating a new proliferation site, this reporter signal became specifically localized at this site (Fig 6I–6K), aligning with the DR5 activity pattern observed during de novo meristem initiation (Fig 3B–3E). At 9 DAT, after the male-to-hermaphrodite transition had been completed and the de novo meristem was fully established, *CrTAA1* reporter expression was explicitly localized at the center of the newly formed meristem (Fig 6L and 6M), similar to the expression pattern observed during normal hermaphrodite development (Fig 6D and 6G).

### Stable gene-editing demonstrates the crucial role of *CrTAA1* in male-to-hermaphrodite conversion and de novo meristem formation

Given that *CrTAA1* expression is dynamically and specifically localized to the initiation site of newly formed hermaphrodites and subsequently at the meristem notch region (Fig 6), we hypothesized that CrTAA1 is essential for the de novo formation of meristems and conversion from males to hermaphrodites. This hypothesis was tested and supported by results from chemical perturbation experiments using the TAA1-specific inhibitor Kyn (Figs 4, 5, S2–S7), prompting us to further test the hypothesis through genetic perturbation using CRISPR-Cas9-mediated gene editing.

**Fig 7:**
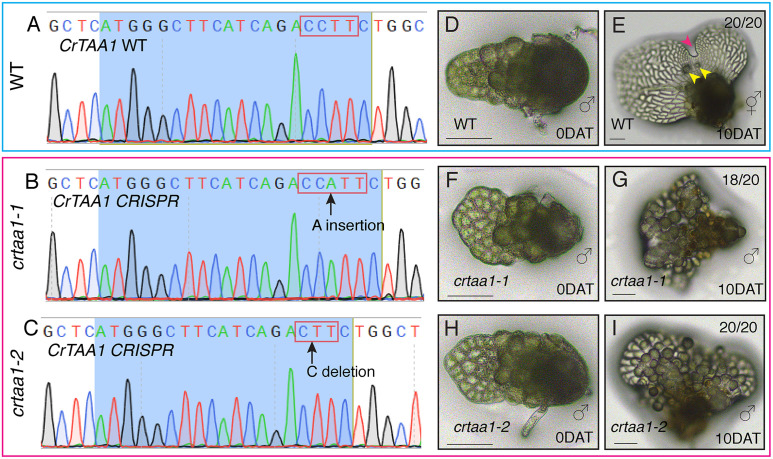
*CrTAA1* is crucial for male-to-hermaphrodite conversion in the absence of antheridiogen. **(A–C)** Sanger sequencing results showing representative WT and edited *CrTAA1* sequences. **(D–I)** Representative micrographs showing male gametophytes (3 DAG) from WT (highlighted in blue) and *CrTAA1-CRISPR* lines (*crtaa1-1* and *crtaa1-2*, highlighted in magenta) transferred from CFM (containing antheridiogen) to antheridiogen-free FM. Images were taken immediately after transfer (0 DAT) (D, F, H) and 10 days after transfer (DAT) (E, G, I), with 20 out of 20 for WT, 18 out of 20 for *crtaa1-1* (line 99), and 20 out of 20 for *crtaa1-2* (line 103). **(E)** Magenta arrowhead: de novo meristem; yellow arrowheads: newly formed archegonia. Scale bars (D–I): 100 µm.

To genetically test the requirement of *CrTAA1*, we generated stable Ceratopteris knockout lines using CRISPR-Cas9. The CRISPR construct was designed with two gRNAs targeting exon 1 of *CrTAA1*, each driven by an independent CrU6 promoter and terminated by a single CrU6 terminator. Following the established procedure for stable transformation and regeneration of transgenic lines, we identified multiple lines with different mutations in *CrTAA1*. Following self-fertilization, confirmed knockout lines were obtained, and their spores were harvested. Two independent *CrTAA1 CRISPR* mutants, *crtaa1-1* with a single nucleotide insertion (A) and *crtaa1-2* with a single nucleotide deletion (C) in exon 1 (Fig 7A–7C), both introduced frameshifts that led to premature termination, generating null alleles (S20 Fig). These mutant lines, together with WT controls, were selected for detailed phenotypic analysis. To evaluate their ability to undergo the male-to-hermaphrodite transition, individual 3-DAG male gametophytes from WT or *CrTAA1* knockout lines (*crtaa1-1* and *crtaa1-2*) were transferred onto antheridiogen-free FM plates, and their development was monitored by light microscopy immediately after transfer and at 10 DAT. At 10 DAT, all WT gametophytes (20 out of 20) had successfully transitioned into hermaphrodites (Fig 7D and 7E). In contrast, at least 90% of gametophytes from both *CrTAA1* knockout lines remained male at 10 DAT (Fig 7F–7I). The genetic knockout of *CrTAA1* phenocopied the inhibitory effects observed with Kyn treatment, providing robust genetic evidence that TAA1-mediated auxin biosynthesis is essential for male-to-hermaphrodite conversion and the associated de novo meristem formation in *Ceratopteris.*

## Discussion

### Auxin peak and male-to-hermaphrodite conversion

Our previous work demonstrated that transferring Ceratopteris males from an antheridiogen-containing to an antheridiogen-free environment triggers a profound developmental switch: cell proliferation is re-initiated at a new site, establishing the MPC lineage and subsequently leading to hermaphrodite formation [9]. However, the molecular basis by which the absence of antheridiogen induces this reprogramming of cell fate remains unclear. This study begins to address this knowledge gap by identifying a key signaling event, localized auxin biosynthesis, that is dynamically activated under antheridiogen-free conditions and is essential for driving male-to-hermaphrodite conversion.

During this transition, an auxin signal maximum appears to define an early step in cell fate re-specification. Upon transfer to antheridiogen-free conditions, DR5 reporter activity in males is strongly and specifically induced at a newly initiated site of cell proliferation (Fig 3B and 3C). This DR5 signal marks the re-entry of cells into the cell cycle (Fig 5A–5C, 5G–5I, and 5M–5O) and persists throughout the formation of the new hermaphrodite (Fig 3B–3P). This new DR5 signal peak (Fig 3B and 3C) coincides spatially and temporally with the localized activation of *CrTAA1* expression shortly after antheridiogen removal (Fig 6I–6K). Furthermore, this early auxin activation event not only serves as a molecular marker for hermaphrodite initiation but also plays a functional role in enabling sex-type conversion. Chemical inhibition of TAA1-dependent auxin biosynthesis by Kyn abolished the formation of the DR5 signal maximum (Fig 4S–4V) and suppressed male-to-hermaphrodite conversion in a concentration-dependent manner (Fig 4A–4J and S1 Data). Lineage-tracing and quantitative cell division analyses revealed that Kyn treatment severely disrupted MPC lineage establishment and subsequent cell proliferation (Figs 5 and S8-S18). Consistently, CRISPR-Cas9-mediated knockout of *CrTAA1* phenocopied these effects (Fig 4A–4J), effectively blocking male-to-hermaphrodite conversion (Fig 7). Together, these results reveal an essential molecular pathway underlying sex-type conversion: the absence of antheridiogen induces a local auxin biosynthesis hotspot that generates a new auxin signal maximum. This auxin maximum is required to initiate the MPC lineage, sustain cell proliferation, and drive de novo meristem formation, which are essential for successful sex-type conversion. Given that DR5 signals are also dynamically present in female reproductive organs (egg-bearing archegonia) during both normal hermaphrodite development and male-to-hermaphrodite conversion (Figs 1 and 3), future studies should determine whether auxin directly promotes female organ formation and defines hermaphrodite identity, or acts indirectly by regulating meristem activity, which in turn triggers archegonium formation in Ceratopteris [2,44].

### Conserved auxin biosynthesis plays context-specific roles in fern gametophytes

Previous genetic studies have shown that local auxin biosynthesis, mediated by the TAA1-YUC pathway, regulates stem cell maintenance and differentiation in both seed plants and seed-free nonvascular plants [23,25,31,46,47]. In seed plants, disruptions of this pathway impairs stem cell activity and identity in both root and shoot meristems [23,25]. For instance, in *Arabidopsis* SAMs, auxin biosynthesis genes are expressed in the epidermal layer, and the *taa1 tar2* double mutant exhibits reduced shoot meristem size compared to WT [25]. In the liverwort *Marchantia*, auxin biosynthesis is localized to the apical meristem region, as indicated by *MpTAA* transcript and IAA accumulation [31]. Loss-of-function *mptaa* mutants display reduced thallus growth and impaired tissue differentiation [31]. In this study, we uncover a previously undefined role for auxin biosynthesis in regulating sex type and de novo meristem formation in fern gametophytes. Our results (S19 Fig and S2 Data), together with previous analysis [39], demonstrate that CrTAA1 (Ceric.10G039800) is the closest homolog of *Arabidopsis* TAA1, whereas other related Ceratopteris proteins group into a distinct clade corresponding to the functionally uncharacterized *Arabidopsis* TAR3/4 (S19 Fig). *CrTAA1* is specifically activated at the onset of the male-to-hermaphrodite transition, and its expression becomes confined to the center of emerging de novo meristems (Fig 6L and 6M), overlapping with the developing concave meristem notch. These patterns align with the crucial role of *CrTAA1* in initiating and establishing new meristems during the haploid gametophyte phase. Consistent with these findings, previous transcriptomic and phylogenetic analyses identified multiple *YUC* homologs in the Ceratopteris genome [42]. One of these homologs is activated during early hermaphrodite development and meristem establishment, dependent on CrHAM, a meristem-specific transcription factor that promotes meristem cell division and prevents male differentiation [42,48]. Moreover, treatment with yucasin [49], a specific inhibitor of YUC family enzymes in both flowering plants (such as *Arabidopsis*) and bryophytes (such as *Physcomitrium* and *Marchantia*) [49,50], effectively blocked de novo meristem formation and male-to-hermaphrodite conversion in Ceratopteris (S21 Fig and S5 Data), similar to the defects caused by chemical or genetic perturbation of *CrTAA1*. Together, these results highlight that the TAA1-YUC-mediated auxin biosynthesis pathway is evolutionarily conserved across land plants, including bryophytes, seed-free vascular plants, and seed plants, yet plays distinct, context-specific roles in fern gametophytes, where it governs de novo meristem formation and defines the early, essential steps of sex-type conversion. Furthermore, because Ceratopteris hermaphrodites and *Marchantia* gametophytes share comparable notch meristems and similar localized expression of their *TAA1* homologs, comparative studies of auxin function in these systems will further illuminate the evolution of meristem regulation in land plants.

Beyond the meristem initiation sites and newly formed meristems observed during sex-type conversion (Fig 3), auxin signals revealed by the DR5 reporter are also active during normal meristem development in hermaphrodites and in differentiated organs, including rhizoids, sperm-producing antheridia, and egg-bearing archegonia (Figs 1 and 2). This suggests multiple and potentially distinct roles of auxin in regulating Ceratopteris gametophyte development across different stages. Future genetic studies using the established *CrTAA1* mutants and other CRISPR-generated auxin-related mutants, focusing on developmental processes such as meristem notch formation in hermaphrodites, as well as antheridium differentiation and spermatogenesis in males, will help establish a more comprehensive understanding of auxin function during the fern gametophyte phase. Furthermore, the DR5 reporter examined in this study reflects the transcriptional output of auxin signaling, based on ARF gene activity [41]. Although no previous studies have applied it in ferns, the ratiometric auxin sensor R2D2 has been shown across multiple plant lineages to serve as a direct readout for auxin input or accumulation [41]. Future studies using R2D2 as an independent reporter system to determine auxin dynamics in Ceratopteris gametophytes, in direct comparison with DR5 expression patterns, will provide more insights into auxin function during fern development.

## Materials and methods

### Plant material and growth condition

Plant material and growth condition in this study followed the procedures described in Geng and colleagues (2022) [44]. *Ceratopteris richardii* (Ceratopteris) strain Hn-n [3] was used as the WT for generating transgenic plants. Ceratopteris gametophytes were grown on either fern media (FM) containing 0.5 × MS salts and vitamins (PhytoTechnology Laboratories) and 0.7% (w/v) agar (Sigma-Aldrich) or conditioned FM (CFM, supplemented with antheridiogen) as previously described [5]. Hermaphrodites were individually transferred to 48-well plates for self-fertilization. *Ceratopteris calli* were induced from young sporophytes (shoot tips or fronds) on callus induction medium (pH 5.8) containing 1 × MS salts and vitamins (PhytoTechnology Laboratories), 2% (w/v) sucrose, 1 mg/L benzylaminopurine, and 0.7% agar (Sigma–Aldrich). Gametophytes, calli, and sporophytes were cultured under continuous light at 29 °C. Fertilized sporophytes or regenerated sporophytic shoots were transferred to soil. Adult sporophytes were subsequently transferred to large soil pots and cultivated in the Purdue LILY greenhouse for spore harvesting.

### Plasmid construct and transformation

The *DR5v2::ntdTomato* reporter was generated by PCR amplifying the *DR5v2::ntdTomato* fragment from the *pGreen-DR5v1::3xGFP-DR5v2::tdtomato* [41], followed by cloning into the pCAMBIA1300 vector using PstI and SpeI restriction sites. The ubiquitous nuclear marker, *pCrUBQ10::H2B-GFP::3′CrUBQ10* (line 10), used in this study, was previously described [9]. To generate the *CrTAA1* transcriptional reporter (*pCrTAA1::H2B-GFP::3′CrTAA1*), a 2,496 base-pair (bp) promoter and 1,466 bp 3′ terminator of *CrTAA1* were introduced into the 5′ and 3′ ends of the *H2B-GFP* fragment, respectively, in the pENTR vector using enzyme restriction and ligation. The *CrTAA1* promoter was amplified from the Ceratopteris genome using the primers: 5′-ACAAGCGGCCGCTTCGAGGACCTAGACCCGAGACTTC-3′ and 5′-ACAAGCGGCCGCAGCTACTACAGCAAAACGCTTAACC-3′. The 3′ terminator of *CrTAA1* was amplified using the primers: 5′-TTGGCGCGCCTCGACAGATAAGTTTTGTTCATTCG-3′ and 5′-TTGGCGCGCCTCAATACATGCTCGCTGGGCATTAAG-3′. The entire expression cassette (*pCrTAA1::H2B-GFP::3′CrTAA1*) was subsequently cloned into the pMOA34 vector for stable transformation.

The CRISPR construct for *Ceratopteris* was generated using the template from a previously published system for *Arabidopsis* [51]. Specifically, the modified system contains the *CrUBQ10* promoter [9] to drive Cas9 expression and two distinct *Ceratopteris* Pol III promoters to independently drive gRNAs. For editing the *CrTAA1* genomic sequence, two gRNAs specific to *CrTAA1* were designed using the website tool (chopchop.cbu.uib.no), with the *Ceratopteris richardii* genome sequence [46] as the reference. The gRNA1 (5′-AUGGGCUUCAUCAGACCUUC-3′) and gRNA2 (5′-AUAUACUAACUGUUCGGGUC-3′) were positioned 78 bp apart, targeting exon 1 of *CrTAA1*. The expression cassettes for gRNA1 and gRNA2 were independently generated through Q5 PCR mutagenesis (New England Biolabs) in two separate pCR4 plasmids, CRP01 and CRP02, using the following primers: 5′- tcagaccttcGTTTTAGAGCTAGAAATAGCAAGTTAAAATAAGGCTAG-3′ and 5′- tgaagcccatCGAGTGCAAGGACGCGCG-3′ for gRNA1; 5′- tgttcgggtcGTTTTAGAGCTAGAAATAGCAAGTTAAAATAAG-3′ and 5′-gttagtatatCAACAGCAGCCAATCGCT-3′ for gRNA2. Subsequently, the two gRNA expression cassettes were integrated into a new pCR4 plasmid (CRP03) by enzyme restriction and ligation. The complete gRNA expression cassettes (shown in S22 Fig), along with the *pCrUBQ10::Cas9* expression fragment, were introduced into pCAMBIA1300 for stable transformation.

All constructs were transformed into *Ceratopteris* Hn-n calli using microparticle bombardment, following the previously described procedure [48] Plasmid DNA was coated onto tungsten microparticles and delivered into *Ceratopteris calli* using the BioRad Biolistic PDS-1000/He particle delivery system at 1,100 psi. Transformed calli were cultured on selection media containing Hygromycin (40 µg/mL) for shoot regeneration. Spores (T1) were harvested from each regenerated sporophyte (T0) and tested again on selection media containing 18 µg/ml Hygromycin B. For fluorescent reporters, multiple independent transgenic lines were generated for each construct, showing comparable expression patterns. All live-imaging experiments were performed on FM or CFM without any selection pressure. For initially screening *CrTAA1-CRISPR* mutants, leaves from regenerated sporophytes derived from transformed calli were collected for genomic DNA isolation and subsequent genotyping of *CrTAA1*. Spores from edited sporophytes were then harvested, surface-fertilized, and germinated to produce gametophytes for further genotyping. Individual gametophytes were self-fertilized in 48-well plates, and the resulting sporophytes were genotyped again. *CrTAA1* CRISPR mutants confirmed by Sanger sequencing were used for phenotypic characterization. Chromatographs of Sanger sequencing results were visualized using SnapGene Viewer.

### Effects of chemical and genetic perturbations of auxin biosynthesis on male-to-hermaphrodite conversion

*Ceratopteris* spores were surface-sterilized and spread on CFM to induce male differentiation. At 3 DAG, individual male gametophytes were transferred to FM plates (one gametophyte per plate) lacking antheridiogen to induce male-to-hermaphrodite conversion. Live light micrographs of gametophytes were captured at 0, 7, and 10 days after transfer (DAT) using an Olympus CKX53 microscope equipped with a MIchrome 5 Pro camera under a 10x objective lens. To assess the effects of L-kynurenine, a TAA1 inhibitor, on male-to-hermaphrodite conversion, 3-DAG WT male gametophytes were transferred onto fresh FM plates (without antheridiogen), supplemented with either 0.15% Dimethylsulfoxide (DMSO, as a mock control), 50 µM L-kynurenine (with 0.15% DMSO), or 150 µM L-kynurenine (with 0.15% DMSO). The sex type of each gametophyte was determined at 10 DAT, and the sex ratio for each treatment group was calculated as the average of three independent groups, each consisting of 20 independent biological replicates (for a total of 60 individual gametophytes). To assess the genetic role of *CrTAA1* in this process, gametophytes from two *CrTAA1-CRISPR* mutant lines (Line 99 and Line 103, corresponding to *crtaa1-1* and *crtaa1-2*, respectively) and the WT control were imaged and compared under the same conditions. At 10 DAT, the sex type of each gametophyte was determined, with 20 independent gametophytes analyzed per genotype. The genotypes of the *CrTAA1*-CRISPR lines were confirmed by PCR using primers 5′-AGACAACTTCAGATGCAAAATGA-3′ and 5′-TACGGCAGGCAACTCTGTAGG-3′ and followed by Sanger sequencing. Additionally, to assess the effects of Yucasin (Sigma), an inhibitor of YUC family enzymes [49], on male-to-hermaphrodite conversion, 3-DAG WT male gametophytes were transferred onto fresh FM plates (without antheridiogen) supplemented with either 0.3% DMSO (mock control) or 150 µM Yucasin in 0.3% DMSO. The sex type of each gametophyte was determined at 10 DAT, with 20 individuals analyzed per treatment group.

### Imaging auxin signaling dynamics

Confocal time-lapse live imaging was performed to examine the dynamic expression patterns of the DR5 reporter in *Ceratopteris* gametophytes under different growth conditions. Spores from *DR5v2::ntdTomato* transgenic lines were surface-sterilized and spread on either FM or CFM. Live imaging was performed every day from 1 to 6 DAG. Hermaphrodites germinated on FM were imaged directly on their original plates, while male gametophytes germinated on CFM were also imaged directly on CFM plates. To examine DR5 expression during male-to-hermaphrodite conversion, spores were surface-sterilized and spread on CFM to induce male differentiation. At 3 DAG, male gametophytes were transferred to FM (one gametophyte per plate), and time-lapse confocal imaging was performed at 0 h after transfer (HAT) and every 12 h from 48 to 216 HAT. tdTomato signals were excited with a 561-nm laser line, and emissions was collected within 561–650 nm range. To assess the effects of L-kynurenine on DR5 expression dynamics during male-to-hermaphrodite conversion, 3-DAG male gametophytes were transferred to FM lacking antheridiogen but supplemented with either 0.15% DMSO (mock control) or 150 µM L-kynurenine containing 0.15% DMSO. Confocal images were taken at 1 DAT and at 24-h intervals from 3 to 9 DAT. To examine the dynamic response of this DR5 reporter to auxin, 3-DAG gametophytes of the *DR5v2::ntdTomato* reporter line were transferred from FM to FM supplemented with either 0.1% ethanol (mock control) or 10 µM IAA containing 0.1% ethanol. Confocal live images were taken at 0 and 24 h after treatment using identical settings. To analyze *CrTAA1* expression patterns during hermaphrodite development, spores from *Ceratopteris pCrTAA1::H2B-GFP::3′CrTAA1* transgenic lines were surface-sterilized and cultured on FM plates. Reporter signals were imaged at various DAG in different hermaphrodite samples. To examine *CrTAA1* expression patterns during male-to-hermaphrodite conversion, the *pCrTAA1::H2B-GFP::3′CrTAA1* spores were spread on CFM to induce male differentiation. At 3 DAG, individual male gametophyte were transferred to an antheridiogen-free FM plate (one sample per plate) to initiate the conversion process. Reporter signals were examined at 3 and 9 DAT in different samples. GFP was excited using a 488-nm laser line, with the detection wavelength set to 490–535 nm.

### Reverse transcription-quantitative polymerase chain reaction (RT-qPCR) analysis

The expression pattern of *CrTAA1* (Ceric.10G039800) in WT male gametophytes (MG) and hermaphroditic gametophytes (HG) was determined by qPCR (Fig 6A and S4 Data). *Ceratopteris* WT spores were surface-sterilized and spread on FM plates. HG and MG were separately collected from the same WT populations at 4, 5, 8, or 10 DAG. Each biological replicate consisted of pooled HG or MG samples, and at least three biological replicates per stage were used for RNA isolation and qPCR analysis. The procedure for culturing, harvesting gametophytes, RNA isolation, and qPCR followed the previously described methods [42]. All gametophytes used for qPCR were cultured in growth chambers (Percival) under continuous light at 29 °C. Total RNA was extracted using the RNeasy Mini Kit (Qiagen), and cDNA was synthesized using the SuperScript Reverse Transcription system (Invitrogen). qPCR was performed using SYBR Green qPCR Master Mix (Selleck Chemicals) on a CFX duet Real-time PCR system (Bio-Rad). Relative expression levels of *CrTAA1* were normalized to the internal control *CrACTIN1.* The qPCR primers for *CrACTIN1* were 5′-GAGAGAGGCTACTCTTTCACAACC-3′ and 5′-AGGAAGTTCGTAACTCTTCTCCAA-3′ [42]. The qPCR primers for *CrTAA1* were 5′-ACAGCTGAGACATGCAATCGG-3′ and 5′-GACAGCATGACAGTCTTCATCC-3′.

### Confocal time-lapse imaging during male-to-hermaphrodite conversion with auxin biosynthesis inhibitor treatment

Spores of *pCrUBQ10::H2B-GFP::3′CrUBQ10* were surface-sterilized and spread on CFM to induce male differentiation. At 2 DAG, male gametophytes were transferred to FM lacking antheridiogen but supplemented with either 0.15% DMSO (mock control) or 150 µM L-kynurenine containing 0.15% DMSO. To avoid potential effects of newly released antheridiogen, only one gametophyte was placed per plate. Time-lapse confocal live imaging was performed every 6 h from 0 to 120 HAT. GFP signals were excited with a 488-nm laser line, and emissions were collected within a 490–535 nm detection range. At the final time point (120 h), gametophytes were stained with propidium iodide (PI) to visualize cell outlines, as described previously [9,44,52]. PI was excited using a 561 nm laser line, and emissions were collected in the 596–650 nm range. Confocal image stacks were processed using Fiji/ Image J software to generate maximum intensity z-projection views.

All confocal imaging of *Ceratopteris* gametophytes was performed using a Zeiss LSM 880 upright confocal microscope with a Plan Apochromat 10×/0.45 objective lens. When necessary, the brightness of each entire confocal image was uniformly adjusted in Fiji/ImageJ to improve visualization.

### Lineage tracking and cell division quantification

Nucleus segmentation, lineage tracking, and cell division quantification were performed following the detailed procedure described previously [9,44].

### Identification and phylogenetic analysis of *TAA1* homologs in *Ceratopteris*

The identification and phylogenetic analysis of *TAA1/TAR1/TAR2* homologs in *Ceratopteris richardii* were performed as follows: Protein sequences of *Arabidopsis* AtTAA1 and AtTAR homologs were used as queries for BLASTp searches against the genomes of *Ceratopteris* and two additional fern species, *Azolla filiculoides and Salvinia cucullata*. BLASTp searches were performed using Phytozome 13 (phytozome-next.jgi.doe.gov) and Fernbase (https://fernbase.org/), with an E-value threshold set at 10^−16^. Previously published TAA1 and TAR homologs from selected species (listed in S3 Data) were obtained from Carrillo-Carrasco and colleagues [45] via the Phytozome 13 database. Multiple sequence alignments were carried out using MAFFT (https://www.ebi.ac.uk/jdispatcher/msa/mafft), and maximum-likelihood phylogenetic trees were constructed using IQ-TREE v2.4 (http://www.iqtree.org/) with 100,000 ultrafast bootstrap replicates for statistical support. The phylogenetic tree was visualized using Figtree (http://tree.bio.ed.ac.uk/software/figtree/). Protein domain identification for each clade was performed using INTERPROSCAN [53], which was also used to confirm the identities of protein domains in shorter BLASTp hits (less than 200 amino acids) that passed the *E*-value threshold.

## Supporting information

S1 Fig DR5 signals in Ceratopteris gametophytes in response to exogenous IAA. **(A–P)** Confocal time-lapse images of Ceratopteris gametophytes expressing the *DR5v2::ntdTomato* reporter. Images of 3-DAG gametophytes, either hermaphrodite (A, I) or male (C, K), were taken at 0 hour (h) and 24 h with either mock treatment (0.1% of ethanol, A–H) or 10 µM IAA (I–P). (A–D, I–L) Z-projection views of tdTomato signals (Fire LUT). (E–H, M–P) Merged channels of tdTomato (magenta) and DIC (gray, showing cell outlines). Color bars (A–D, I–L) represent the relative intensity of DR5 signals in Fire LUT images. Scale bars: 50 µm.
(JPG)

S2 Fig Time-lapse live confocal imaging of the male gametophyte sample 1 in the absence of antheridiogen with mock treatment. **(A–U)** Confocal images of a 2 DAG Ceratopteris male gametophyte expressing *CrUBQ10p::H2B-GFP::3′CrUBQ10*, taken every 6 h from 0 to 120 h after treatment with the mock. Green: GFP. **(V)** Merged channels of GFP (green) and propidium iodide (PI) (magenta). The sample was stained with PI at 120 h to visualize cell outlines. (A) A magenta arrow at 0 h indicates the meristem progenitor cell (MPC). (A–K) Dashed white circles highlight representative antheridia at various time points from initiation to rupture. (M–V) Magenta arrowheads indicate the de novo formation of a meristem. (N) White arrowheads indicate motile sperm released from ruptured antheridia. Scale bars (A–V): 50 µm. Panels (A–V) are all the time points captured from 0 to 120 h for sample 1 (mock-treated) shown in Fig 5A–5C. Specifically, panel (A) is the full image of the zoomed-in region shown in Fig 5A (0 h), panel (K) is the full image for the zoomed-in region shown in Fig 5B (60 h), and panel (V) is the full image for the zoomed-in region shown in Fig 5C (120 h). Three independent biological replicates were imaged under the same conditions, yielding comparable results. Time-lapse confocal imaging results of the other two samples are included in S3 and S4 Figs, respectively.
(JPG)

S3 Fig Time-lapse live confocal imaging of the male gametophyte sample 2 in the absence of antheridiogen with mock treatment. **(A–U)** Confocal images of a 2 DAG Ceratopteris male gametophyte expressing *CrUBQ10p::H2B-GFP::3′CrUBQ10*, taken every 6 h from 0 to 120 h after treatment with the mock. Green: GFP. **(V)** Merged channels of GFP (green) and PI (magenta). The sample was stained with PI at 120 h to visualize cell outlines. (A) A magenta arrow at 0 h indicates the meristem progenitor cell (MPC). (A–I) Dashed white circles highlight representative antheridia at various time points from initiation to rupture. (K–V) Magenta arrowheads indicate the de novo formation of a meristem. Scale bars (A–V): 50 µm. Three independent biological replicates were imaged under the same conditions, yielding comparable results. Time-lapse confocal imaging results of the other two samples are included in S2 and S4 Figs, respectively.
(JPG)

S4 Fig Time-lapse live confocal imaging of the male gametophyte sample 3 in the absence of antheridiogen with mock treatment. **(A–U)** Confocal images of a 2 DAG Ceratopteris male gametophyte expressing *CrUBQ10p::H2B-GFP::3′CrUBQ10*, taken every 6 h from 0 to 120 h with the mock. Green: GFP. (A) A magenta arrow at 0 h indicates the MPC. (A–M) Dashed white circles highlight representative antheridia at various time points from initiation to rupture. (M–U) Magenta arrowheads indicate the de novo formation of a meristem. Scale bars (A–U): 50 µm. Three independent biological replicates were imaged under the same conditions, yielding comparable results. Time-lapse confocal imaging results of the other two samples are included in S2 and S3 Figs, respectively.
(JPG)

S5 Fig Time-lapse live confocal imaging of the male gametophyte sample 4 in the absence of antheridiogen but in the presence of an auxin biosynthesis inhibitor. **(A–U)** Confocal images of a 2 DAG Ceratopteris male gametophyte expressing *CrUBQ10p::H2B-GFP::3′CrUBQ10*, taken every 6 h from 0 to 120 h after treatment (HAT) with 150 µM L-kyneurine (Kyn). Green: GFP. **(V)** Merged channels of GFP (green) and propidium iodide (PI) (magenta). The sample was stained with PI at 120 h to visualize cell outlines. (A–N) Dashed white circles highlight representative antheridia at various time points from initiation to rupture. Scale bars (A–V): 50 µm. Panels (A–V) are all the time points captured from 0 to 120 h for sample 4 (Kyn-treated) shown in Fig 5D–5F. Specifically, panel (A) is the full image of the zoomed-in region shown in Fig 5D (0 h), panel (K) is the full image for the zoomed-in region shown in Fig 5E (60 h), and panel (V) is the full image for the zoomed-in region shown in Fig 5F (120 h). Three independent biological replicates were imaged under the same conditions, yielding comparable results. Time-lapse confocal imaging results of the other two samples are included in S6 and S7 Figs, respectively.
(JPG)

S6 Fig Time-lapse live confocal imaging of the male gametophyte sample 5 in the absence of antheridiogen but in the presence of an auxin biosynthesis inhibitor. **(A–U)** Confocal images of a 2 DAG Ceratopteris male gametophyte expressing *CrUBQ10p::H2B-GFP::3′CrUBQ10*, taken every 6 h from 0 to 120 h with 150 µM L-kyneurine. Green: GFP. **(V)** Merged channels of GFP (green) and PI (magenta). The sample was stained with PI at 120 h to visualize cell outlines. (A–N) Dashed white circles highlight representative antheridia at various time points from initiation to rupture. Scale bars (A–V): 50 µm. Three independent biological replicates were imaged under the same conditions, yielding comparable results. Time-lapse confocal imaging results of the other two samples are included in S5 and S7 Figs, respectively.
(JPG)

S7 Fig Time-lapse live confocal imaging of the male gametophyte sample 6 in the absence of antheridiogen but in the presence of an auxin biosynthesis inhibitor. **(A–U)** Confocal images of a 2 DAG Ceratopteris male gametophyte expressing *CrUBQ10p::H2B-GFP::3′CrUBQ10*, taken every 6 h from 0 to 120 h with 150 µM L-kyneurine. Green: GFP. **(V)** Merged channels of GFP (green) and PI (magenta). The sample was stained with PI at 120 h to visualize cell outlines. (A–N) Dashed white circles highlight representative antheridia at various time points from initiation to rupture. Scale bars (A–V): 50 µm. Three independent biological replicates were imaged under the same conditions, yielding comparable results. Time-lapse confocal imaging results of the other two samples are included in S5 and S6 Figs, respectively.
(JPG)

S8 Fig Cell lineage dynamics of the live-imaged male gametophyte sample 1 in the absence of antheridiogen with mock treatment. **(A–U)** Lineage maps of the gametophyte imaged from 0 to 120 h (shown in S2 Fig). Each solid circle represents either a single nucleus or a group of nuclei from one antheridium. At 0 h, adjacent nuclei were labeled with different colors as a reference for visualizing distinct lineages at subsequent time points. Cells and their progeny were labeled with the same color across all time points to represent the same lineage. When an antheridium developed into a 3D complex structure, all nuclei within the same antheridium were represented as a single solid circle to simplify visualization. A magenta arrow in (A) highlights the meristem progenitor cell (MPC, yellow) lineage, which contributes to de novo meristem formation. Scale bars (A–U): 50 µm. Panels (A–U) are the complete cell lineage maps analyzed from 0 to 120 h for sample 1 (mock-treated) shown in Fig 5G–5I. Specifically, panel (A) is the full image of the zoomed-in region shown in Fig 5G (0 h), panel (K) is the full image for the zoomed-in region shown in Fig 5H (60 h), and panel (U) is the full image for the zoomed-in region shown in Fig 5I (120 h). Lineages from three independent samples were analyzed, showing comparable results. Dynamic cell lineage maps of the other two samples are included in S9 and S10 Figs, respectively.
(JPG)

S9 Fig Cell lineage dynamics of the live-imaged male gametophyte sample 2 in the absence of antheridiogen with mock treatment. **(A–U)** Lineage maps of the gametophyte imaged from 0 to 120 h (shown in S3 Fig). Each solid circle represents either a single nucleus or a group of nuclei from one antheridium. At 0 h, adjacent nuclei were labeled with different colors as a reference for visualizing distinct lineages at subsequent time points. Cells and their progeny were labeled with the same color across all time points to represent the same lineage. When an antheridium developed into a 3D complex structure, all nuclei within the same antheridium were represented as a single solid circle to simplify visualization. A magenta arrow in (A) highlights the MPC (yellow) lineage, which contributes to de novo meristem formation. Scale bars (A–U): 50 µm. Lineages from three independent samples were analyzed, showing comparable results. Dynamic cell lineage maps of the other two samples are included in S8 and S10 Figs, respectively.
(JPG)

S10 Fig Cell lineage dynamics of the live-imaged male gametophyte sample 3 in the absence of antheridiogen with mock treatment. **(A–U)** Lineage maps of the gametophyte imaged from 0 to 120 h (shown in S4 Fig). Each solid circle represents either a single nucleus or a group of nuclei from one antheridium. At 0 h, adjacent nuclei were labeled with different colors as a reference for visualizing distinct lineages at subsequent time points. Cells and their progeny were labeled with the same color across all time points to represent the same lineage. When an antheridium developed into a 3D complex structure, all nuclei within the same antheridium were represented as a single solid circle to simplify visualization. A magenta arrow in (A) highlights the MPC (yellow) lineage, which contributes to de novo meristem formation. Scale bars (A–U): 50 µm. Lineages from three independent samples were analyzed, showing comparable results. Dynamic cell lineage maps of the other two samples are included in S8 and S9 Figs, respectively.
(JPG)

S11 Fig Cell lineage dynamics of the live-imaged male gametophyte sample 4 in the absence of antheridiogen with L-kyneurine treatment. **(A–U)** Lineage maps of the gametophyte imaged from 0 to 120 h (shown in S5 Fig). Each solid circle represents either a single nucleus or a group of nuclei within an antheridium. At 0 h, adjacent nuclei were labeled with different colors as a reference for visualizing distinct lineages at subsequent time points. Cells and their progeny were labeled with the same color across all time points to represent the same lineage. When an antheridium developed into a 3D complex structure, all nuclei within the same antheridium were represented as a single solid circle to simplify visualization. A magenta arrow in (A) highlights the cell lineage (yellow), which underwent the most division events during the analyzed period. Scale bars (A–U): 50 µm. Panels (A–U) are the complete cell lineage maps analyzed from 0 h to 120 h for sample 4 (Kyn-treated) shown in Fig 5J–5L. Specifically, panel (A) is the full image of the zoomed-in region shown in Fig 5J (0 h), panel (K) is the full image for the zoomed-in region shown in Fig 5K (60 h), and panel (U) is the full image for the zoomed-in region shown in Fig 5L (120 h). Lineages from three independent samples were analyzed, showing comparable results. Dynamic cell lineage maps of the other two samples are included in S12 and S13 Figs, respectively.
(JPG)

S12 Fig Cell lineage dynamics of the live-imaged male gametophyte sample 5 in the absence of antheridiogen with L-kyneurine treatment. **(A–U)** Lineage maps of the gametophyte imaged from 0 to 120 h (shown in S6 Fig). Each solid circle represents either a single nucleus or a group of nuclei within an antheridium. At 0 h, adjacent nuclei were labeled with different colors as a reference for visualizing distinct lineages at subsequent time points. Cells and their progeny were labeled with the same color across all time points to represent the same lineage. When an antheridium developed into a 3D complex structure, all nuclei within the same antheridium were represented as a single solid circle to simplify visualization. A magenta arrow in (A) highlights the cell lineage (yellow), which underwent the most division events during the analyzed period. Scale bars (A–U): 50 µm. Lineages from three independent samples were analyzed, showing comparable results. Dynamic cell lineage maps of the other two samples are included in S11 and S13 Figs, respectively.
(JPG)

S13 Fig Cell lineage dynamics of the live-imaged male gametophyte sample 6 in the absence of antheridiogen with L-kyneurine treatment. **(A–U)** Lineage maps of the gametophyte imaged from 0 to 120 h (shown in S7 Fig). Each solid circle represents either a single nucleus or a group of nuclei within an antheridium. At 0 h, adjacent nuclei were labeled with different colors as a reference for visualizing distinct lineages at subsequent time points. Cells and their progeny were labeled with the same color across all time points to represent the same lineage. When an antheridium developed into a 3D complex structure, all nuclei within the same antheridium were represented as a single solid circle to simplify visualization. A magenta arrow in (A) highlights the cell lineage (yellow), which underwent the most division events during the analyzed period. Scale bars (A–U): 50 µm. Lineages from three independent samples were analyzed, showing comparable results. Dynamic cell lineage maps of the other two samples are included in S11 and S12 Figs, respectively.
(JPG)

S14 Fig Comparison of cell division dynamics during 12-h intervals in gametophytes treated with mock or L-kynurenine. Each solid circle represents a single nucleus from the confocal images (S2 and S5 Figs). When an antheridium developed into a 3D complex structure, it was represented as a single solid circle for clear visualization, and subsequent division events within the antheridium were excluded from the quantitative analysis. **(A–T)** Magenta solid circles indicate cells that underwent division, while green solid circles indicate cells that remained undivided during the indicated 12-h period. (A–D, I–K, O–Q) Yellow dashed outlines highlight the MPC lineage in the mock-treated sample 1. (F–H, L–N, R–T) White dashed outlines indicate the most actively dividing cell lineage in the Kyn-treated sample 4 over time. Magenta arrowheads (O–Q) indicate the de novo formation of a multicellular meristem in the mock-treated sample. Scale bar (T): 50 µm, applicable to all individual panels. Three independent samples were analyzed, showing comparable results for each treatment. Cell division maps for mock-treated samples 2 and 3 are included in S15 and S16 Figs, respectively, while those for the other two L-kynurenine-treated samples (Samples 5 and 6) are included in S17 and S18 Figs, respectively.
(JPG)

S15 Fig Cell division dynamics during each 12-h interval in the male gametophyte sample 2 treated with mock. Each solid circle represents a single nucleus from the confocal images (S3 Fig). When an antheridium developed into a 3D complex structure, it was represented as a single solid circle for clear visualization, and subsequent division events within the antheridium were not included from the quantitative analysis. **(A–J)** Magenta solid circles indicate cells that underwent division, while green solid circles indicate cells that remained undivided during the indicated 12-h period. (A–J) Yellow dashed outlines highlight the MPC lineage over time. Magenta arrowheads (F–J) indicate the de novo formation of a multicellular meristem. Scale bar (J): 50 µm, applicable to all individual panels. Three independent samples were analyzed, showing comparable results. Cell division maps for the other two samples are included in S14 and S16 Figs, respectively.
(JPG)

S16 Fig Cell division dynamics during each 12-h interval in the male gametophyte sample 3 treated with mock. Each solid circle represents a single nucleus from the confocal images (S4 Fig). When an antheridium developed into a 3D complex structure, it was represented as a single solid circle for clear visualization, and subsequent division events within the antheridium were not included from the quantitative analysis. **(A–J)** Magenta solid circles indicate cells that underwent division, while green solid circles indicate cells that remained undivided during the indicated 12-h period. (A–J) Yellow dashed outlines highlight the MPC lineage over time. Magenta arrowheads (F–J) indicate the de novo formation of a multicellular meristem. Scale bar (J): 50 µm, applicable to all individual panels. Three independent samples were analyzed, showing comparable results. Cell division maps for the other two samples are included in S14 and S15 Figs, respectively.
(JPG)

S17 Fig Cell division dynamics during each 12-h interval in the male gametophyte sample 5 treated with L-kynurenine. Each solid circle represents a single nucleus from the confocal images (S6 Fig). When an antheridium developed into a 3D complex structure, it was represented as a single solid circle for clear visualization, and subsequent division events within the antheridium were not included from the quantitative analysis. **(A–J)** Magenta solid circles indicate cells that underwent division, while green solid circles indicate cells that remained undivided during the indicated 12-h period. (A–J) White dashed outlines highlight the most actively dividing cell lineage over time. Scale bar (J): 50 µm, applicable to all individual panels. Three independent samples were analyzed, showing comparable results. Cell division maps for the other two samples are included in S14 and S18 Figs, respectively.
(JPG)

S18 Fig Cell division dynamics during each 12-h interval in the male gametophyte sample 6 treated with L-kynurenine. Each solid circle represents a single nucleus from the confocal images (S7 Fig). When an antheridium developed into a 3D complex structure, it was represented as a single solid circle for clear visualization, and subsequent division events within the antheridium were not included from the quantitative analysis. **(A–J)** Magenta solid circles indicate cells that underwent division, while green solid circles indicate cells that remained undivided during the indicated 12-h period. (A–J) White dashed outlines highlight the most actively dividing cell lineage over time. Scale bar (J): 50 µm, applicable to all individual panels. Three independent samples were analyzed, showing comparable results. Cell division maps for the other two samples are included in S14 and S17 Figs, respectively.
(JPG)

S19 Fig Phylogeny of TAA1-TAR family proteins in land plants. The maximum likelihood tree was constructed using IQ-TREE v2.4 with the JTT+I+R5 model and visualized using Figtree. The tree is midpoint-rooted. The TAA1/TAR1/2 clade is indicated by red lines, while the TAR3/4 clade is indicated by blue lines. Bootstrap values greater than 75 are shown, indicating well-supported branches. The sole TAA1 homolog in Ceratopteris (CrTAA1) is highlighted with a black oval.
(JPG)

S20 Fig Alignment of CrTAA1 amino acid sequences. Protein sequences of CrTAA1 from the wild type and the mutant alleles *crtaa1-1* (line 99) and *crtaa1-2* (line 103) were aligned using Clustal Omega Multiple Sequence Alignment v1.2.4 (https://www.ebi.ac.uk/jdispatcher/msa/clustalo). Asterisks indicate the same amino acids, while dashes indicate missing or altered amino acids in the two *CrTAA1* mutants.
(PDF)

S21 Fig Inhibition of YUC-mediated auxin biosynthesis blocks male-to-hermaphrodite conversion in the absence of antheridiogen. Light micrographs of representative male gametophytes at 0, 7, and 10 days after transfer (DAT) to antheridiogen-free FM, treated with either mock **(A–C)** or 150 µM Yucasin **(D–F)**. In the mock-treated sample, white dashed rectangles in (B, C) indicate the original male body observed in (A). Magenta arrowheads (B, C) mark the newly formed meristem, yellow arrowheads (B, C) indicate newly formed archegonia, and blue arrowheads (A, D–F) highlight several antheridia. (G) The percentage of the successfully converted hermaphrodites was calculated from 20 independent gametophytes per treatment. Scale bars: 100 µm (A–F). The data underlying panel (G) can be found in S5 Data.
(JPG)

S22 Fig DNA sequences of the expression cassettes of gRNAs specific for *CrTAA1.* (PDF)

S1 Data Data underlying Fig 4J. Numbers and percentages of converted hermaphrodites under mock or Kyn treatment.
(XLSX)

S2 Data Protein domain analysis of CrTAA1 and its homologs. Protein domains of CrTAA1 and its homologs in Ceratopteris richardii were analyzed and compared with the five *Arabidopsis* TAA1 and TAR homologs. Protein sequences and IDs were retrieved from Phytozome (https://phytozome-next.jgi.doe.gov/), and analyzed using the InterPro database (www.ebi.ac.uk/interpro/search/sequence/). Green colors indicate members of the TAA1 and TAR1/2 subgroup, which participate in canonical IAA biosynthesis through the IPyA pathway, while blue colors indicate members of the TAR3/4 subgroup.
(XLSX)

S3 Data List of TAA1/TAR homologs included in the phylogenetic analysis. IDs of TAA1/TAR homologs, their corresponding species and lineages, and sequence sources are provided. Protein sequences of *Arabidopsis* AtTAA1 and AtTAR homologs were used as quires in BLASTp searches against the genomes of Ceratopteris and two other fern species, *Azolla filiculoides* and *Salvinia cucullata*. Previously published TAA1 and TAR homologs of selected species were based on Carrillo-Carrasco and colleagues (2023) [45] and obtained via the Phytozome 13 database.
(XLSX)

S4 Data Data underlying Fig 6A. Relative expression levels of *CrTAA1* in hermaphroditic gametophytes (HG) and male gametophytes (MG) at the indicated DAG.
(XLSX)

S5 Data Data underlying S21G Fig. Numbers and percentage of converted hermaphrodites at 10 DAT under mock or Yucasin treatment. (XLSX)

## Abbreviations

CFM: conditioned fern medium
DAG: days after germination
DAT: days after transfer
DMSO: dimethylsulfoxide
FM: fern medium
HAT: hours after transfer
HG: hermaphroditic gametophytes
IAA: indole-3-acetic acid
IPA: indole-3-pyruvic acid
MG: male gametophytes
MPC: meristem progenitor cell
PI: propidium iodide
qPCR: quantitative PCR
TAA1: TRYPTOPHAN AMINOTRANSFERASE OF ARABIDOPSIS 1
WT: wild-type
