# Supplementary figures and images for: Dynamic auxin maxima regulate male-to-hermaphrodite conversion and de novo meristem formation in the fern Ceratopteris gametophytes
Source: PLoS Biol. 2026 Jan 23;24(1):e3003592. doi: 10.1371/journal.pbio.3003592 (PMC12829780; doi:10.1371/journal.pbio.3003592)

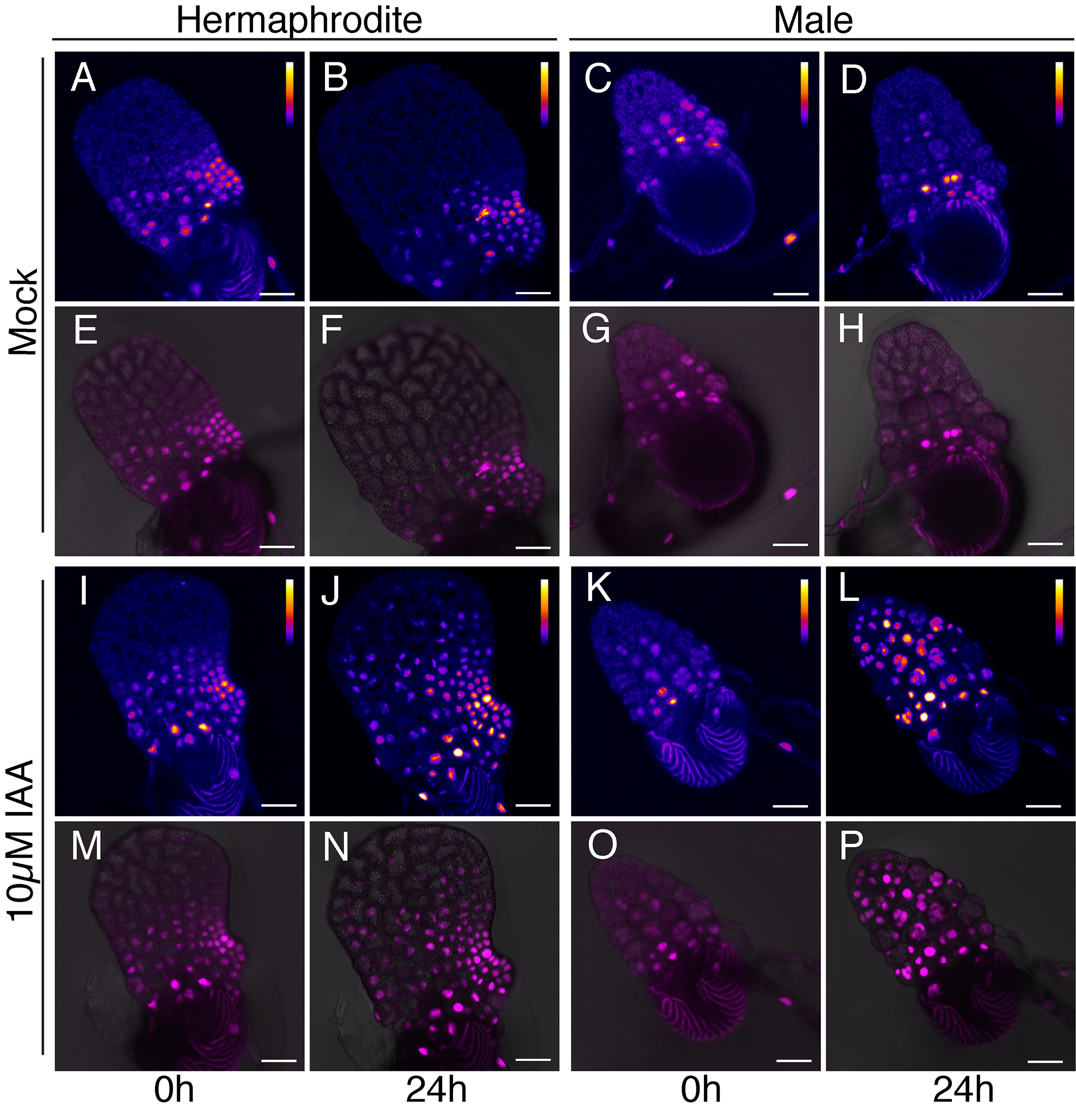

Supplement: S1 Fig — (A–P) Confocal time-lapse images of Ceratopteris gametophytes expressing the DR5v2::ntdTomato reporter. Images of 3-DAG gametophytes, either hermaphrodite (A, I) or male (C, K), were taken at 0 hour (h) and 24 h with either mock treatment (0.1% of ethanol, A–H) or 10 µM IAA (I–P). (A–D, I–L) Z-projection views of tdTomato signals (Fire LUT). (E–H, M–P) Merged channels of tdTomato (magenta) and DIC (gray, showing cell outlines). Color bars (A–D, I–L) represent the relative intensity of DR5 signals in Fire LUT images. Scale bars: 50 µm. (JPG) [file pbio.3003592.s001.jpg]

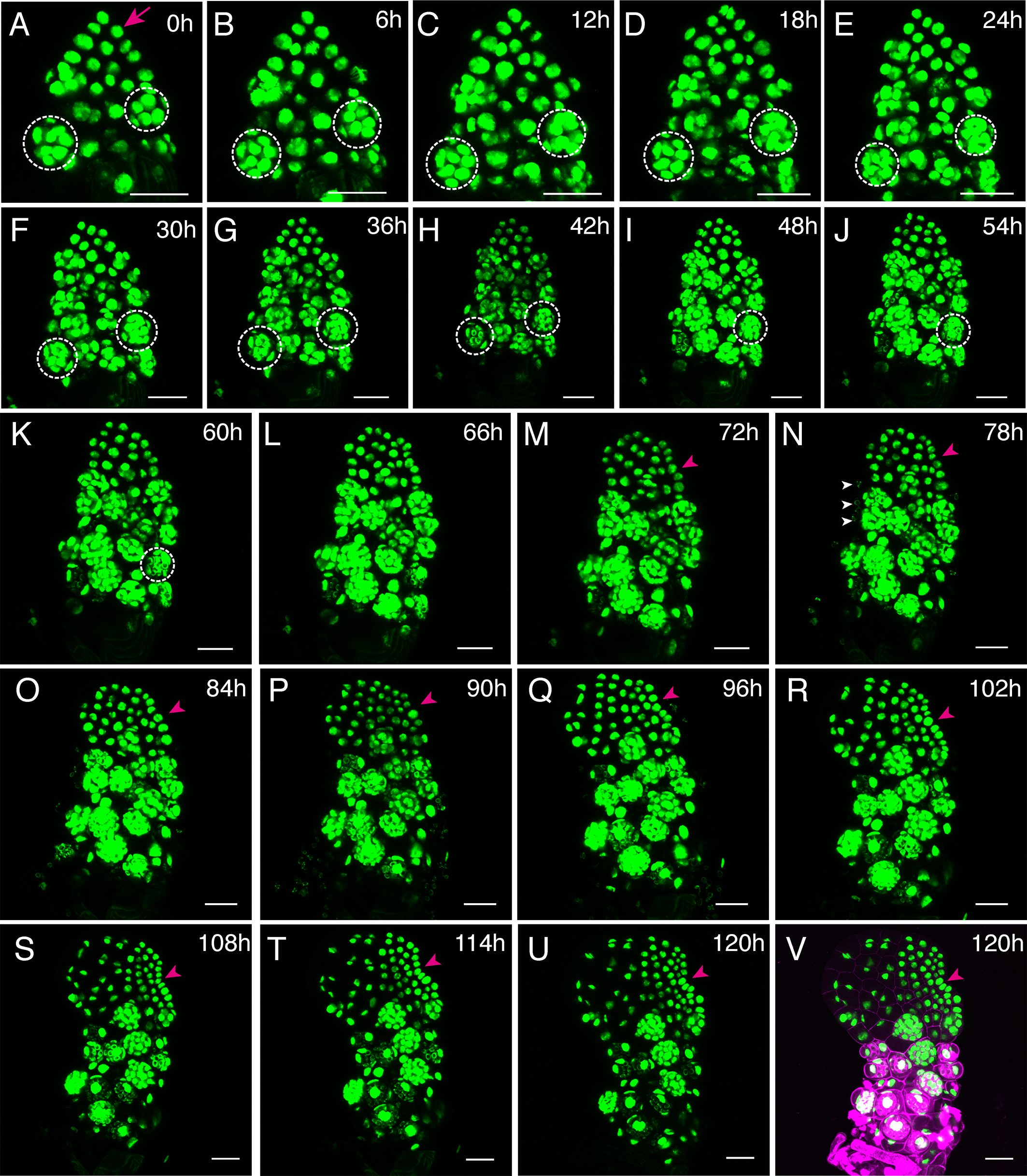

Supplement: S2 Fig — (A–U) Confocal images of a 2 DAG Ceratopteris male gametophyte expressing CrUBQ10p::H2B-GFP::3′CrUBQ10, taken every 6 h from 0 to 120 h after treatment with the mock. Green: GFP. (V) Merged channels of GFP (green) and propidium iodide (PI) (magenta). The sample was stained with PI at 120 h to visualize cell outlines. (A) A magenta arrow at 0 h indicates the meristem progenitor cell (MPC). (A–K) Dashed white circles highlight representative antheridia at various time points from initiation to rupture. (M–V) Magenta arrowheads indicate the de novo formation of a meristem. (N) White arrowheads indicate motile sperm released from ruptured antheridia. Scale bars (A–V): 50 µm. Panels (A–V) are all the time points captured from 0 to 120 h for sample 1 (mock-treated) shown in Fig 5A–5C. Specifically, panel (A) is the full image of the zoomed-in region shown in Fig 5A (0 h), panel (K) is the full image for the zoomed-in region shown in Fig 5B (60 h), and panel (V) is the full image for the zoomed-in region shown in Fig 5C (120 h). Three independent biological replicates were imaged under the same conditions, yielding comparable results. Time-lapse confocal imaging results of the other two samples are included in S3 and S4 Figs, respectively. (JPG) [file pbio.3003592.s002.jpg]

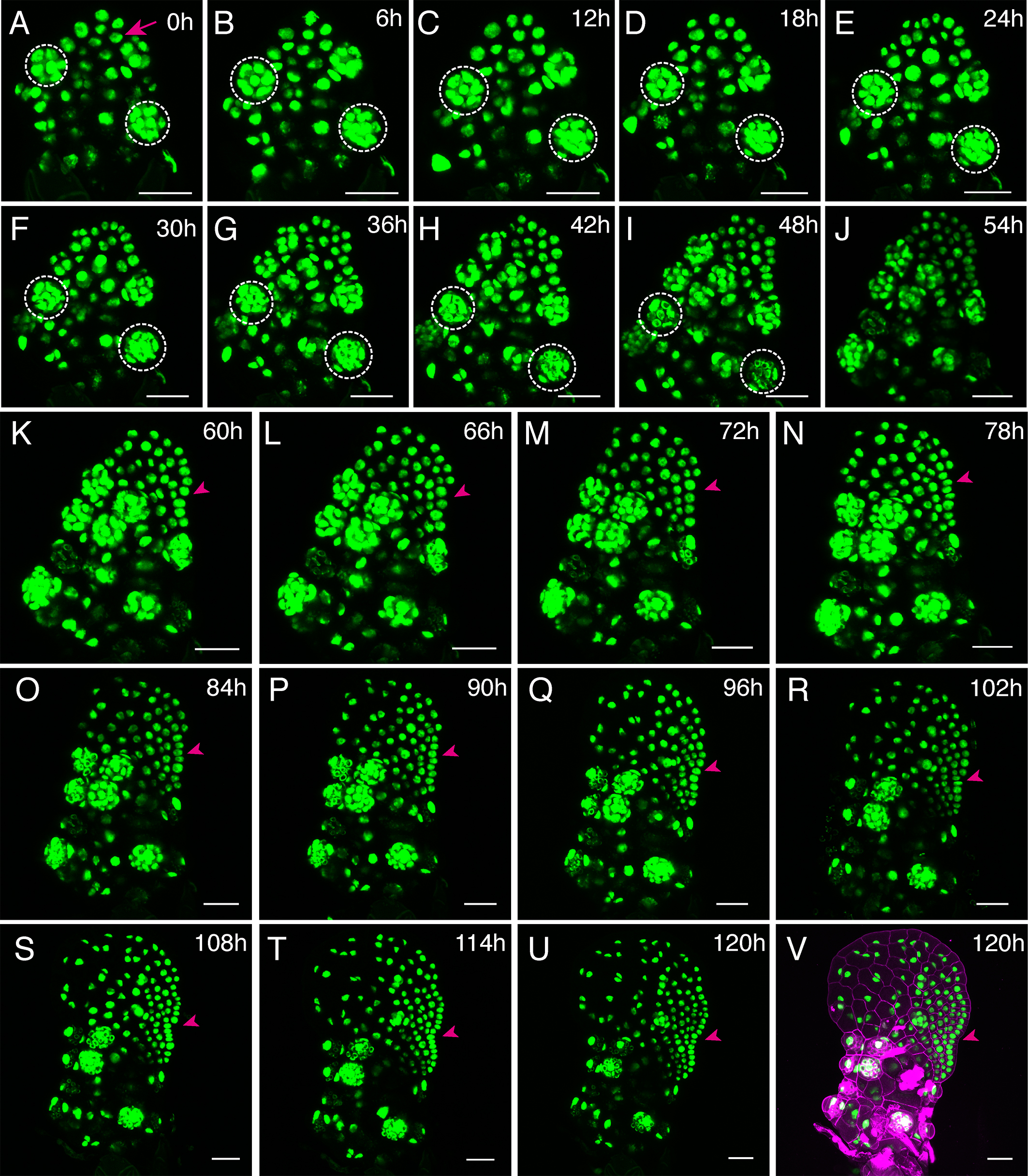

Supplement: S3 Fig — (A–U) Confocal images of a 2 DAG Ceratopteris male gametophyte expressing CrUBQ10p::H2B-GFP::3′CrUBQ10, taken every 6 h from 0 to 120 h after treatment with the mock. Green: GFP. (V) Merged channels of GFP (green) and PI (magenta). The sample was stained with PI at 120 h to visualize cell outlines. (A) A magenta arrow at 0 h indicates the meristem progenitor cell (MPC). (A–I) Dashed white circles highlight representative antheridia at various time points from initiation to rupture. (K–V) Magenta arrowheads indicate the de novo formation of a meristem. Scale bars (A–V): 50 µm. Three independent biological replicates were imaged under the same conditions, yielding comparable results. Time-lapse confocal imaging results of the other two samples are included in S2 and S4 Figs, respectively. (JPG) [file pbio.3003592.s003.jpg]

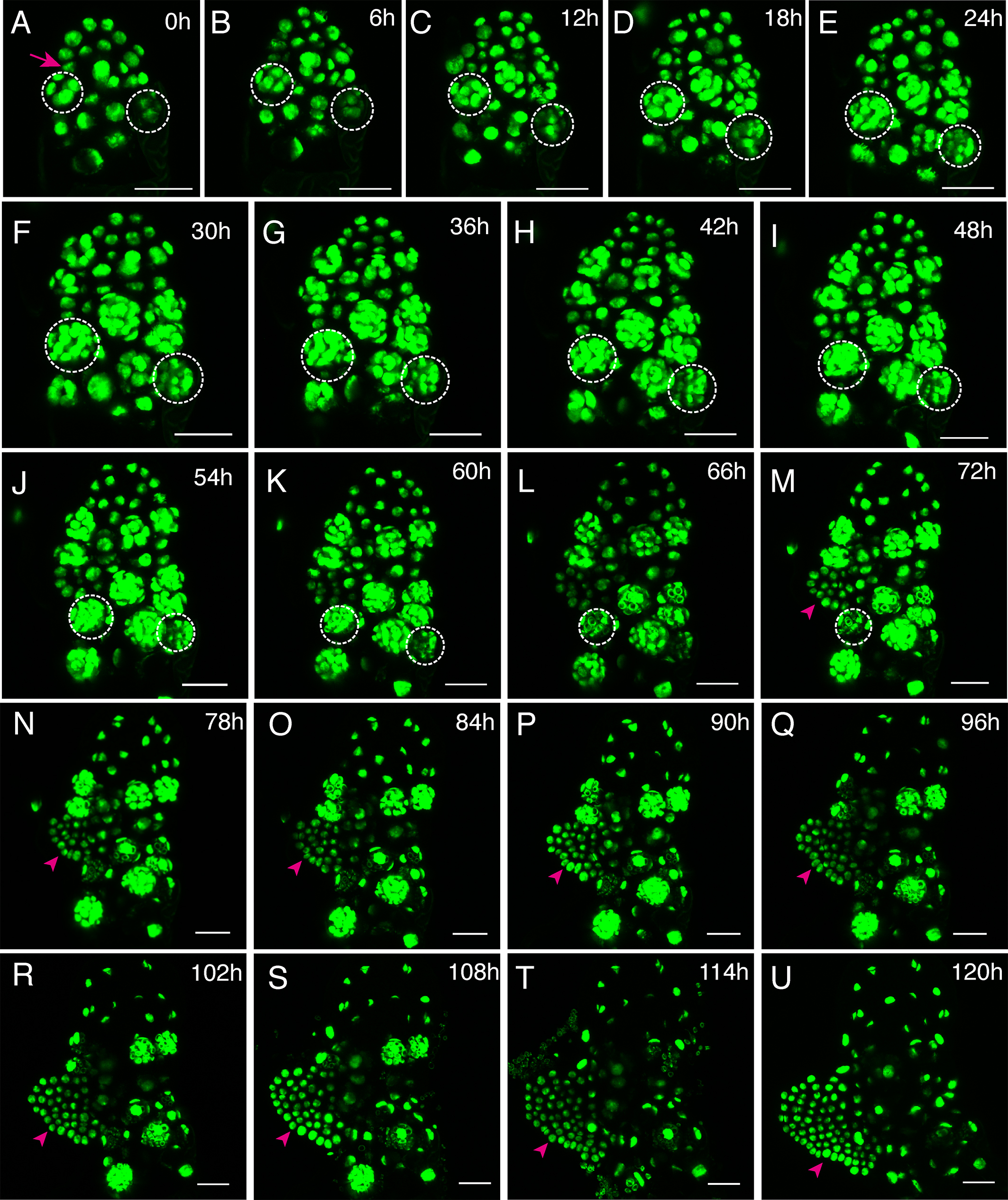

Supplement: S4 Fig — (A–U) Confocal images of a 2 DAG Ceratopteris male gametophyte expressing CrUBQ10p::H2B-GFP::3′CrUBQ10, taken every 6 h from 0 to 120 h with the mock. Green: GFP. (A) A magenta arrow at 0 h indicates the MPC. (A–M) Dashed white circles highlight representative antheridia at various time points from initiation to rupture. (M–U) Magenta arrowheads indicate the de novo formation of a meristem. Scale bars (A–U): 50 µm. Three independent biological replicates were imaged under the same conditions, yielding comparable results. Time-lapse confocal imaging results of the other two samples are included in S2 and S3 Figs, respectively. (JPG) [file pbio.3003592.s004.jpg]

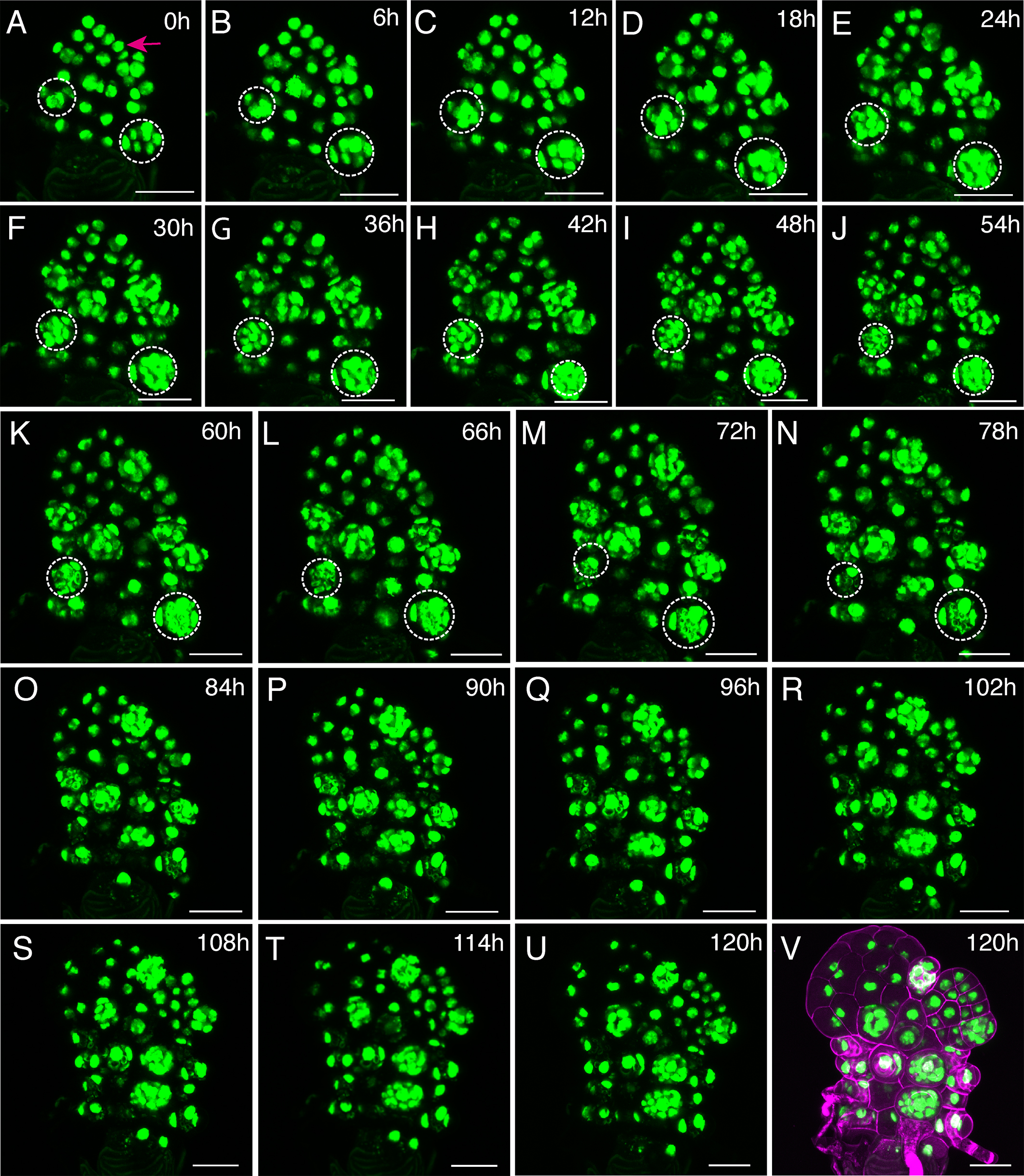

Supplement: S5 Fig — (A–U) Confocal images of a 2 DAG Ceratopteris male gametophyte expressing CrUBQ10p::H2B-GFP::3′CrUBQ10, taken every 6 h from 0 to 120 h after treatment (HAT) with 150 µM L-kyneurine (Kyn). Green: GFP. (V) Merged channels of GFP (green) and propidium iodide (PI) (magenta). The sample was stained with PI at 120 h to visualize cell outlines. (A–N) Dashed white circles highlight representative antheridia at various time points from initiation to rupture. Scale bars (A–V): 50 µm. Panels (A–V) are all the time points captured from 0 to 120 h for sample 4 (Kyn-treated) shown in Fig 5D–5F. Specifically, panel (A) is the full image of the zoomed-in region shown in Fig 5D (0 h), panel (K) is the full image for the zoomed-in region shown in Fig 5E (60 h), and panel (V) is the full image for the zoomed-in region shown in Fig 5F (120 h). Three independent biological replicates were imaged under the same conditions, yielding comparable results. Time-lapse confocal imaging results of the other two samples are included in S6 and S7 Figs, respectively. (JPG) [file pbio.3003592.s005.jpg]

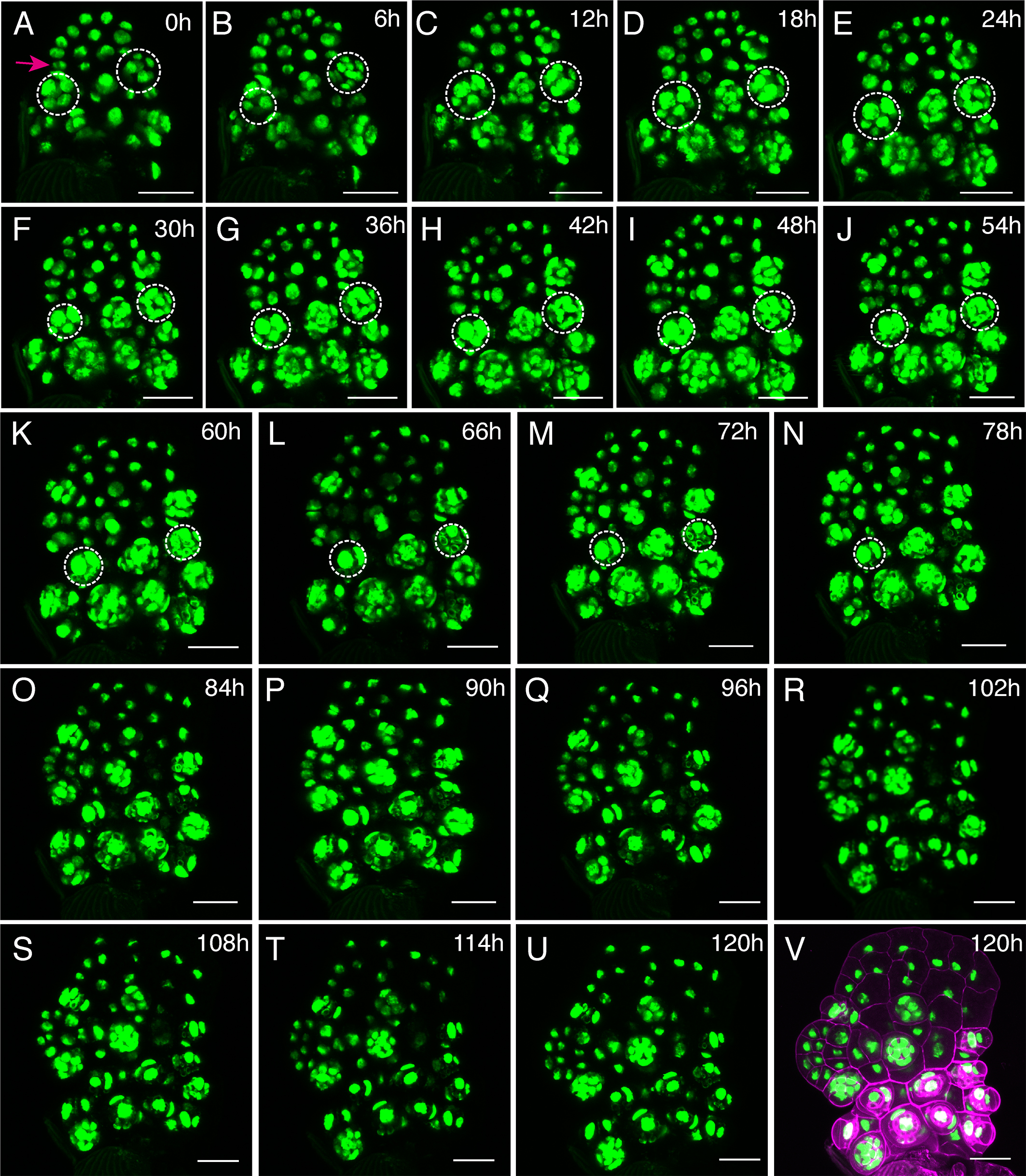

Supplement: S6 Fig — (A–U) Confocal images of a 2 DAG Ceratopteris male gametophyte expressing CrUBQ10p::H2B-GFP::3′CrUBQ10, taken every 6 h from 0 to 120 h with 150 µM L-kyneurine. Green: GFP. (V) Merged channels of GFP (green) and PI (magenta). The sample was stained with PI at 120 h to visualize cell outlines. (A–N) Dashed white circles highlight representative antheridia at various time points from initiation to rupture. Scale bars (A–V): 50 µm. Three independent biological replicates were imaged under the same conditions, yielding comparable results. Time-lapse confocal imaging results of the other two samples are included in S5 and S7 Figs, respectively. (JPG) [file pbio.3003592.s006.jpg]

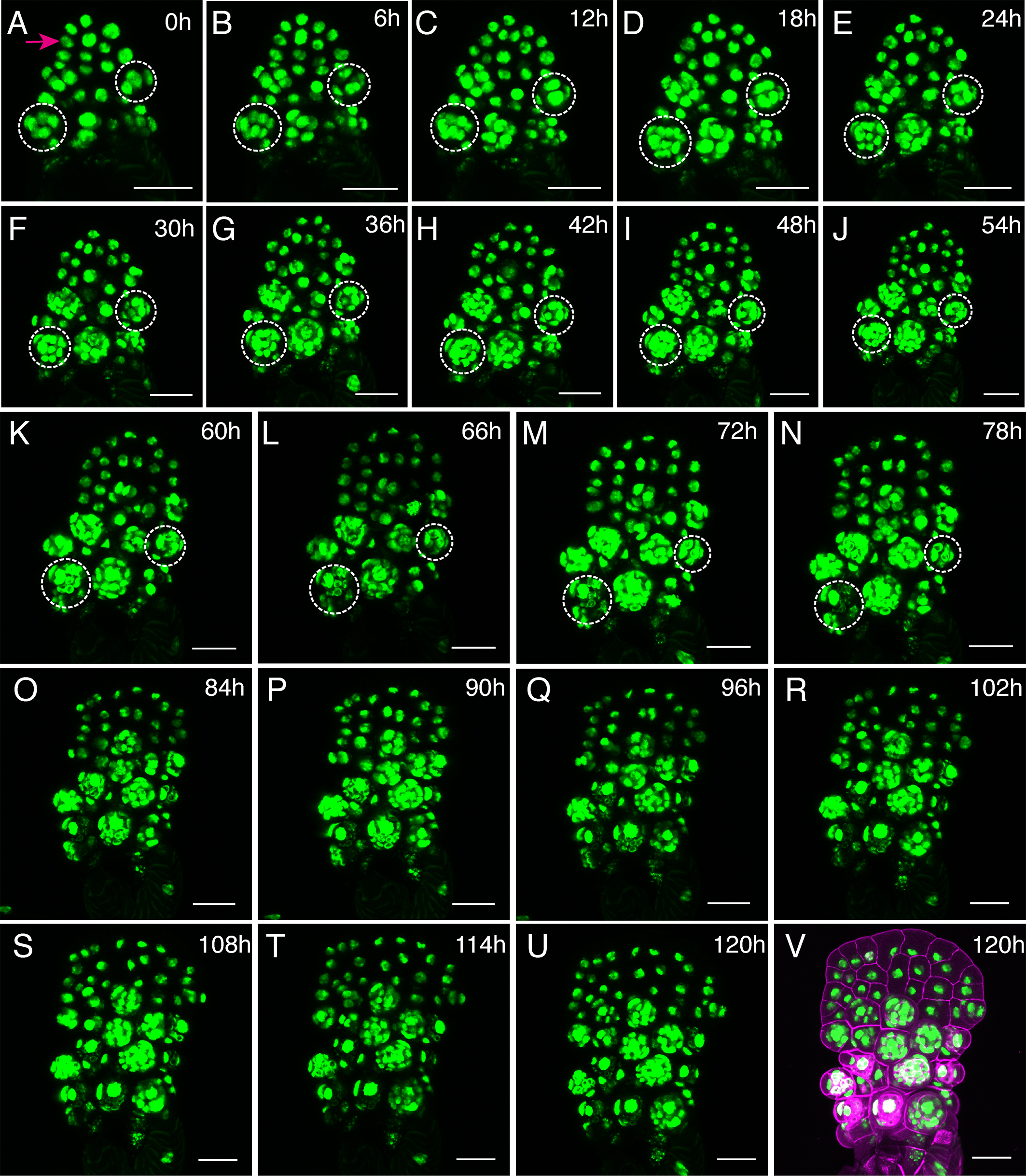

Supplement: S7 Fig — (A–U) Confocal images of a 2 DAG Ceratopteris male gametophyte expressing CrUBQ10p::H2B-GFP::3′CrUBQ10, taken every 6 h from 0 to 120 h with 150 µM L-kyneurine. Green: GFP. (V) Merged channels of GFP (green) and PI (magenta). The sample was stained with PI at 120 h to visualize cell outlines. (A–N) Dashed white circles highlight representative antheridia at various time points from initiation to rupture. Scale bars (A–V): 50 µm. Three independent biological replicates were imaged under the same conditions, yielding comparable results. Time-lapse confocal imaging results of the other two samples are included in S5 and S6 Figs, respectively. (JPG) [file pbio.3003592.s007.jpg]

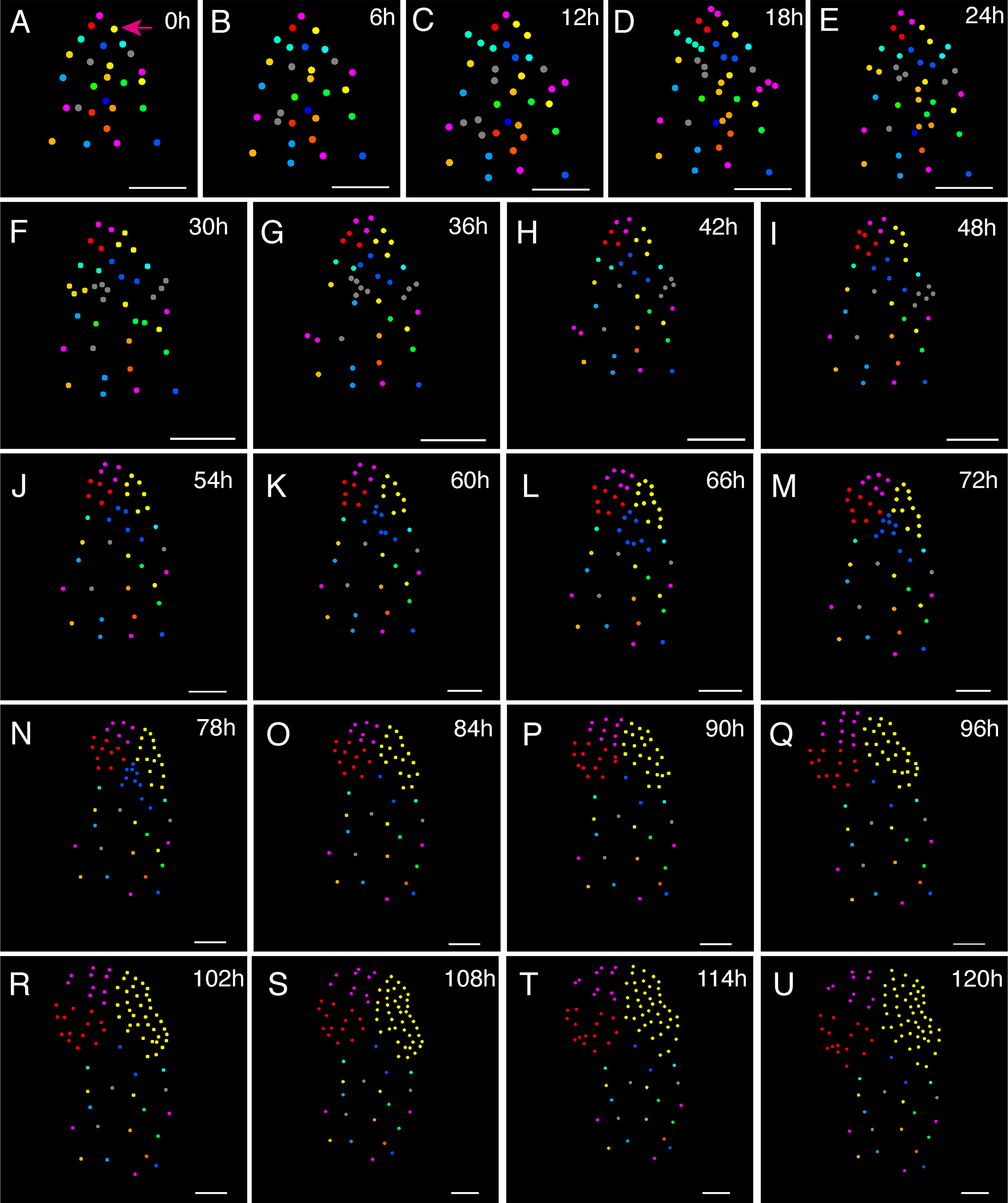

Supplement: S8 Fig — (A–U) Lineage maps of the gametophyte imaged from 0 to 120 h (shown in S2 Fig). Each solid circle represents either a single nucleus or a group of nuclei from one antheridium. At 0 h, adjacent nuclei were labeled with different colors as a reference for visualizing distinct lineages at subsequent time points. Cells and their progeny were labeled with the same color across all time points to represent the same lineage. When an antheridium developed into a 3D complex structure, all nuclei within the same antheridium were represented as a single solid circle to simplify visualization. A magenta arrow in (A) highlights the meristem progenitor cell (MPC, yellow) lineage, which contributes to de novo meristem formation. Scale bars (A–U): 50 µm. Panels (A–U) are the complete cell lineage maps analyzed from 0 to 120 h for sample 1 (mock-treated) shown in Fig 5G–5I. Specifically, panel (A) is the full image of the zoomed-in region shown in Fig 5G (0 h), panel (K) is the full image for the zoomed-in region shown in Fig 5H (60 h), and panel (U) is the full image for the zoomed-in region shown in Fig 5I (120 h). Lineages from three independent samples were analyzed, showing comparable results. Dynamic cell lineage maps of the other two samples are included in S9 and S10 Figs, respectively. (JPG) [file pbio.3003592.s008.jpg]

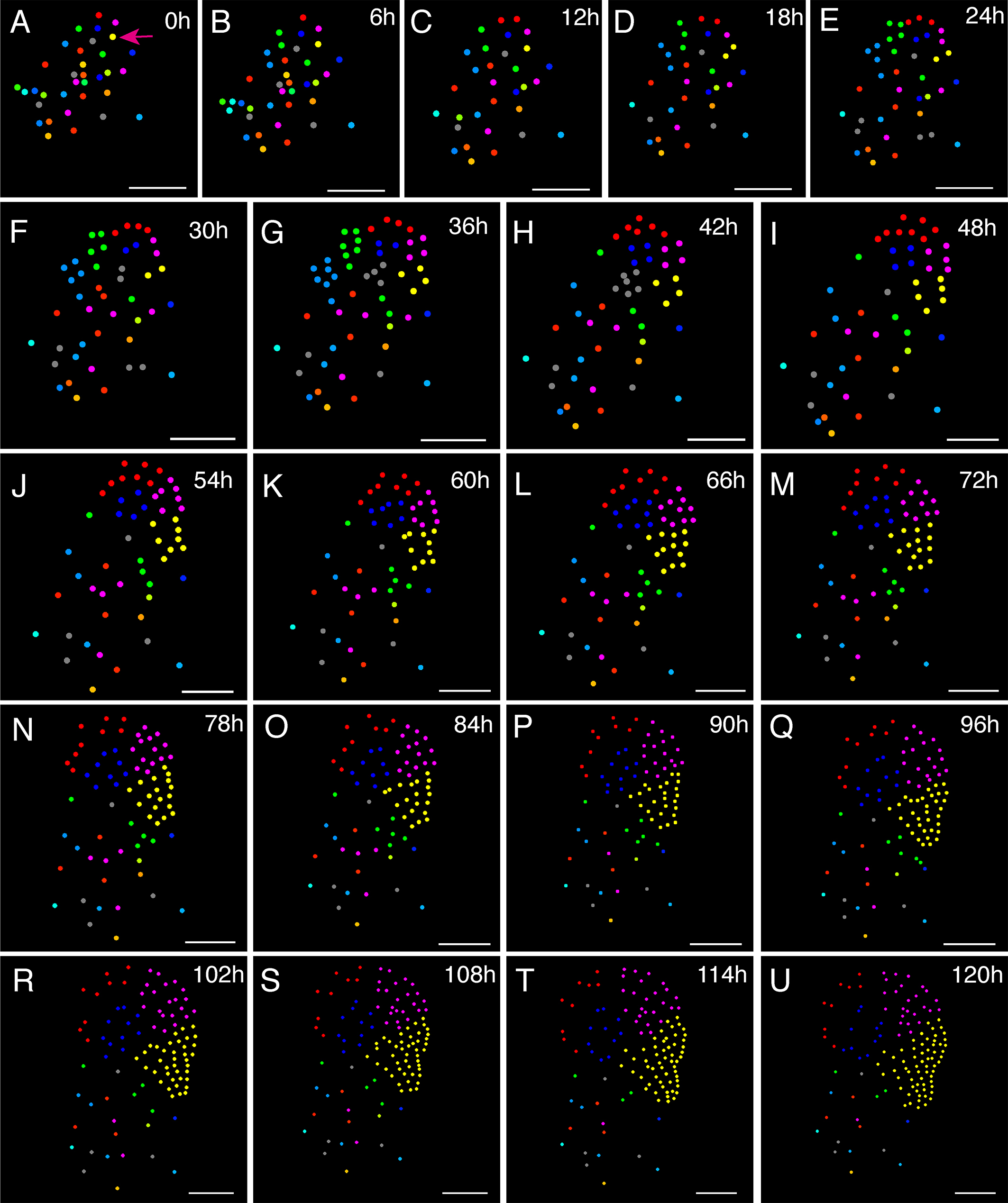

Supplement: S9 Fig — (A–U) Lineage maps of the gametophyte imaged from 0 to 120 h (shown in S3 Fig). Each solid circle represents either a single nucleus or a group of nuclei from one antheridium. At 0 h, adjacent nuclei were labeled with different colors as a reference for visualizing distinct lineages at subsequent time points. Cells and their progeny were labeled with the same color across all time points to represent the same lineage. When an antheridium developed into a 3D complex structure, all nuclei within the same antheridium were represented as a single solid circle to simplify visualization. A magenta arrow in (A) highlights the MPC (yellow) lineage, which contributes to de novo meristem formation. Scale bars (A–U): 50 µm. Lineages from three independent samples were analyzed, showing comparable results. Dynamic cell lineage maps of the other two samples are included in S8 and S10 Figs, respectively. (JPG) [file pbio.3003592.s009.jpg]

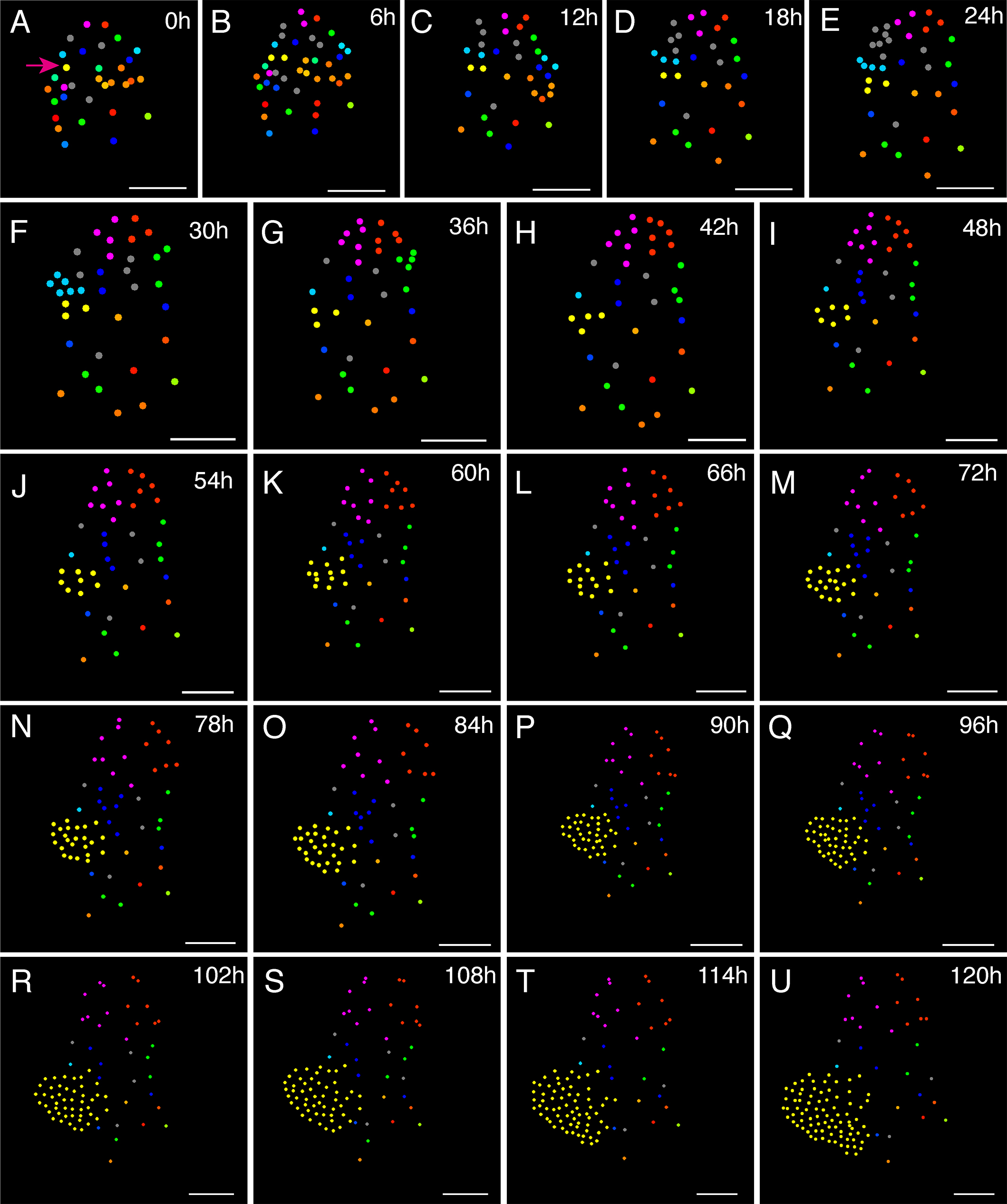

Supplement: S10 Fig — (A–U) Lineage maps of the gametophyte imaged from 0 to 120 h (shown in S4 Fig). Each solid circle represents either a single nucleus or a group of nuclei from one antheridium. At 0 h, adjacent nuclei were labeled with different colors as a reference for visualizing distinct lineages at subsequent time points. Cells and their progeny were labeled with the same color across all time points to represent the same lineage. When an antheridium developed into a 3D complex structure, all nuclei within the same antheridium were represented as a single solid circle to simplify visualization. A magenta arrow in (A) highlights the MPC (yellow) lineage, which contributes to de novo meristem formation. Scale bars (A–U): 50 µm. Lineages from three independent samples were analyzed, showing comparable results. Dynamic cell lineage maps of the other two samples are included in S8 and S9 Figs, respectively. (JPG) [file pbio.3003592.s010.jpg]

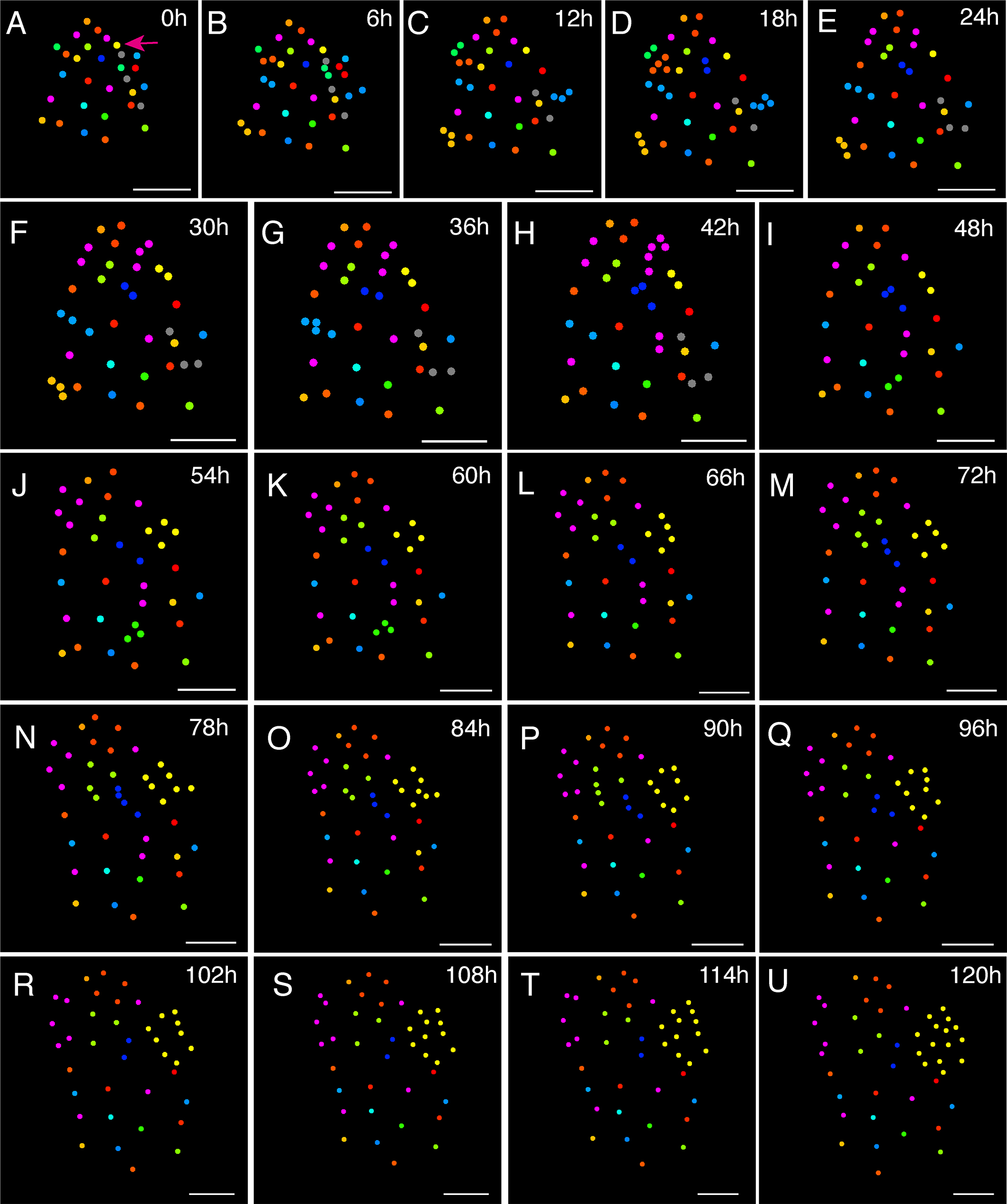

Supplement: S11 Fig — (A–U) Lineage maps of the gametophyte imaged from 0 to 120 h (shown in S5 Fig). Each solid circle represents either a single nucleus or a group of nuclei within an antheridium. At 0 h, adjacent nuclei were labeled with different colors as a reference for visualizing distinct lineages at subsequent time points. Cells and their progeny were labeled with the same color across all time points to represent the same lineage. When an antheridium developed into a 3D complex structure, all nuclei within the same antheridium were represented as a single solid circle to simplify visualization. A magenta arrow in (A) highlights the cell lineage (yellow), which underwent the most division events during the analyzed period. Scale bars (A–U): 50 µm. Panels (A–U) are the complete cell lineage maps analyzed from 0 h to 120 h for sample 4 (Kyn-treated) shown in Fig 5J–5L. Specifically, panel (A) is the full image of the zoomed-in region shown in Fig 5J (0 h), panel (K) is the full image for the zoomed-in region shown in Fig 5K (60 h), and panel (U) is the full image for the zoomed-in region shown in Fig 5L (120 h). Lineages from three independent samples were analyzed, showing comparable results. Dynamic cell lineage maps of the other two samples are included in S12 and S13 Figs, respectively. (JPG) [file pbio.3003592.s011.jpg]

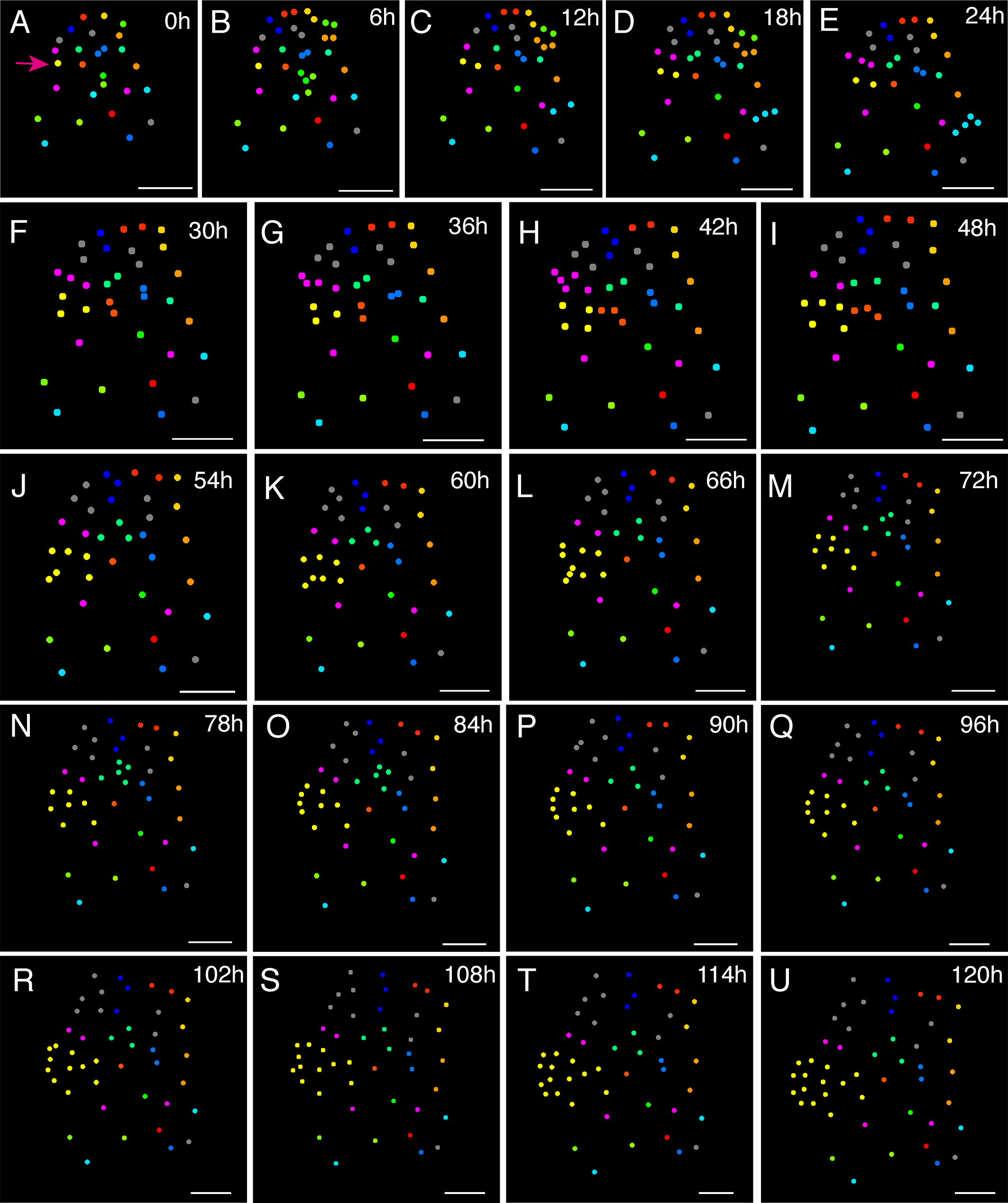

Supplement: S12 Fig — (A–U) Lineage maps of the gametophyte imaged from 0 to 120 h (shown in S6 Fig). Each solid circle represents either a single nucleus or a group of nuclei within an antheridium. At 0 h, adjacent nuclei were labeled with different colors as a reference for visualizing distinct lineages at subsequent time points. Cells and their progeny were labeled with the same color across all time points to represent the same lineage. When an antheridium developed into a 3D complex structure, all nuclei within the same antheridium were represented as a single solid circle to simplify visualization. A magenta arrow in (A) highlights the cell lineage (yellow), which underwent the most division events during the analyzed period. Scale bars (A–U): 50 µm. Lineages from three independent samples were analyzed, showing comparable results. Dynamic cell lineage maps of the other two samples are included in S11 and S13 Figs, respectively. (JPG) [file pbio.3003592.s012.jpg]

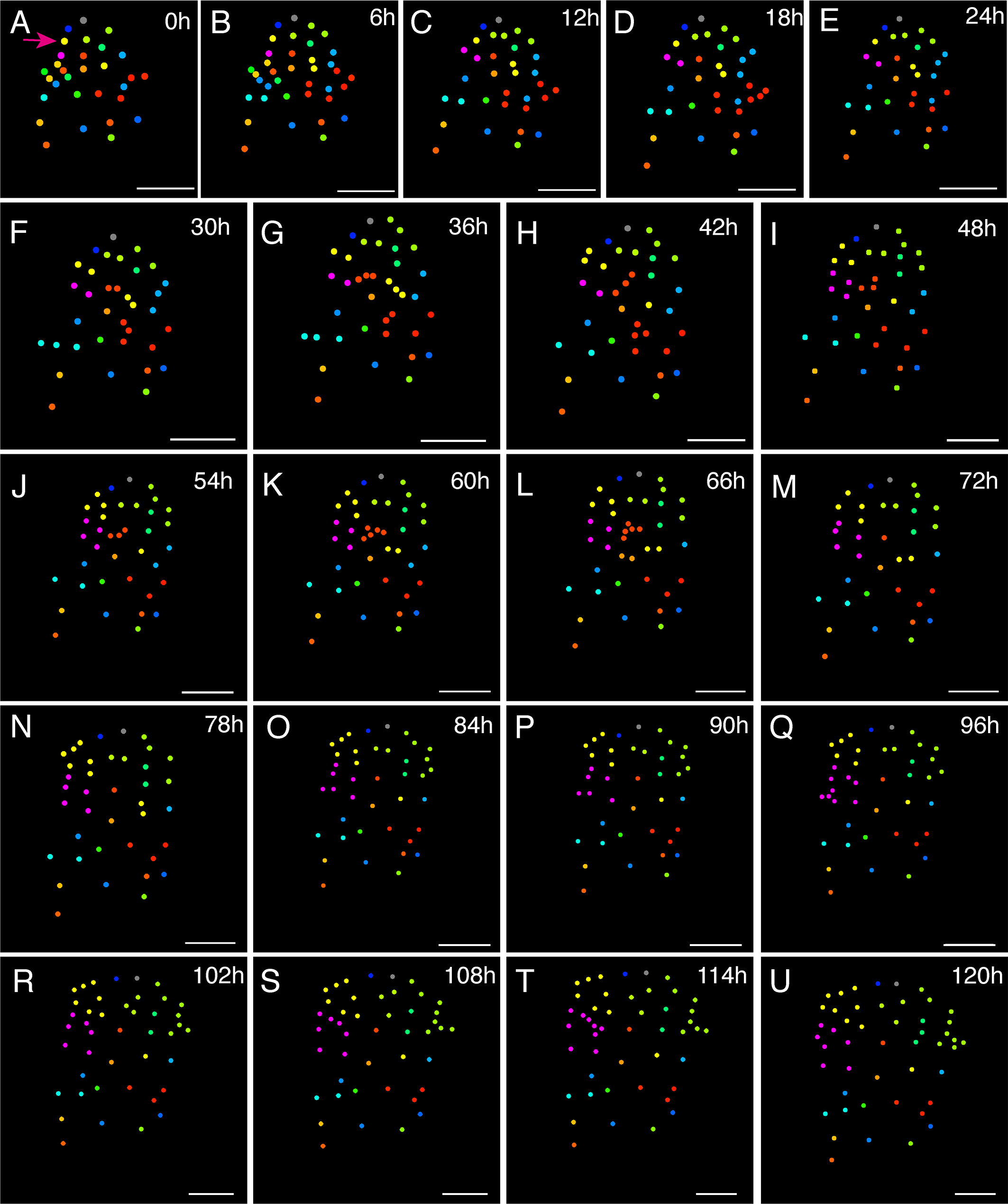

Supplement: S13 Fig — (A–U) Lineage maps of the gametophyte imaged from 0 to 120 h (shown in S7 Fig). Each solid circle represents either a single nucleus or a group of nuclei within an antheridium. At 0 h, adjacent nuclei were labeled with different colors as a reference for visualizing distinct lineages at subsequent time points. Cells and their progeny were labeled with the same color across all time points to represent the same lineage. When an antheridium developed into a 3D complex structure, all nuclei within the same antheridium were represented as a single solid circle to simplify visualization. A magenta arrow in (A) highlights the cell lineage (yellow), which underwent the most division events during the analyzed period. Scale bars (A–U): 50 µm. Lineages from three independent samples were analyzed, showing comparable results. Dynamic cell lineage maps of the other two samples are included in S11 and S12 Figs, respectively. (JPG) [file pbio.3003592.s013.jpg]

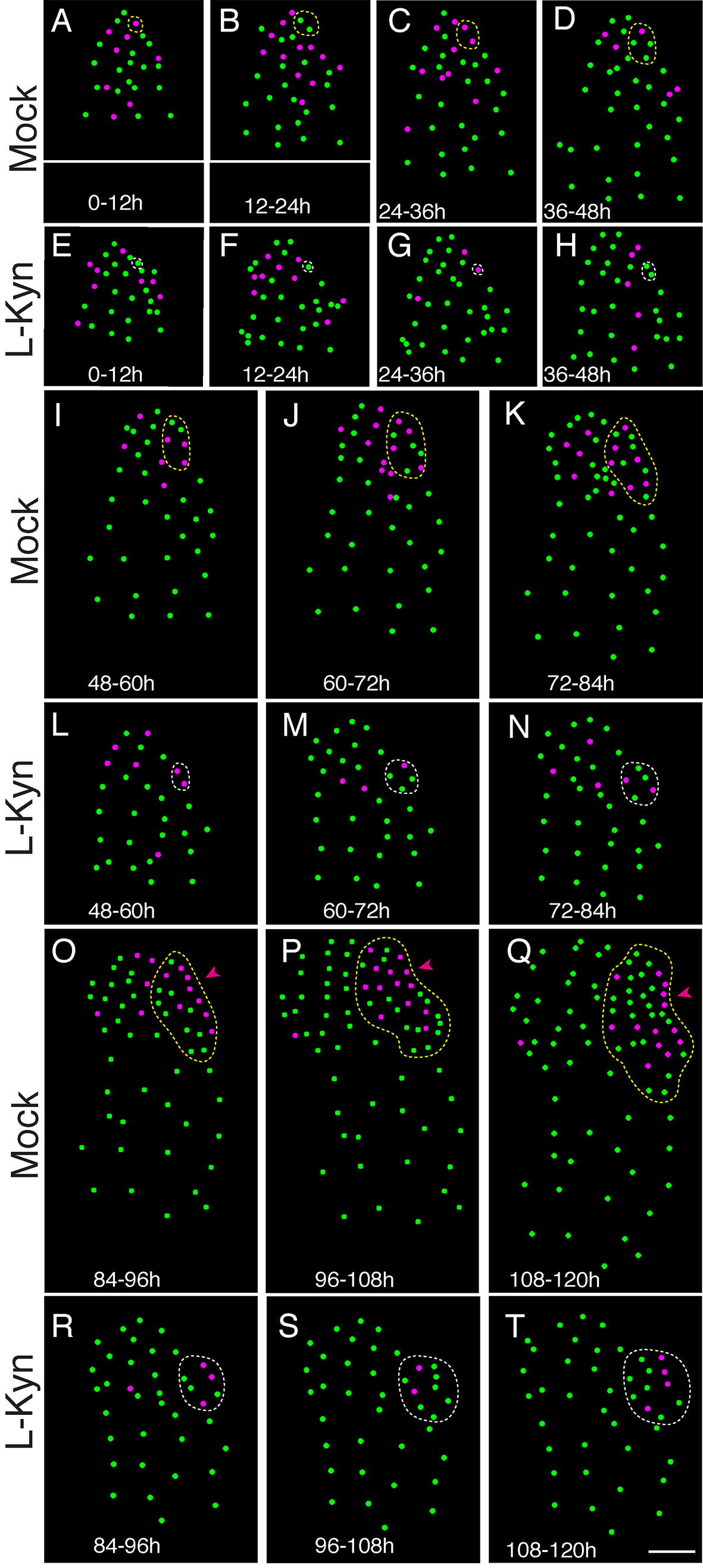

Supplement: S14 Fig — Each solid circle represents a single nucleus from the confocal images (S2 and S5 Figs). When an antheridium developed into a 3D complex structure, it was represented as a single solid circle for clear visualization, and subsequent division events within the antheridium were excluded from the quantitative analysis. (A–T) Magenta solid circles indicate cells that underwent division, while green solid circles indicate cells that remained undivided during the indicated 12-h period. (A–D, I–K, O–Q) Yellow dashed outlines highlight the MPC lineage in the mock-treated sample 1. (F–H, L–N, R–T) White dashed outlines indicate the most actively dividing cell lineage in the Kyn-treated sample 4 over time. Magenta arrowheads (O–Q) indicate the de novo formation of a multicellular meristem in the mock-treated sample. Scale bar (T): 50 µm, applicable to all individual panels. Three independent samples were analyzed, showing comparable results for each treatment. Cell division maps for mock-treated samples 2 and 3 are included in S15 and S16 Figs, respectively, while those for the other two L-kynurenine-treated samples (Samples 5 and 6) are included in S17 and S18 Figs, respectively. (JPG) [file pbio.3003592.s014.jpg]

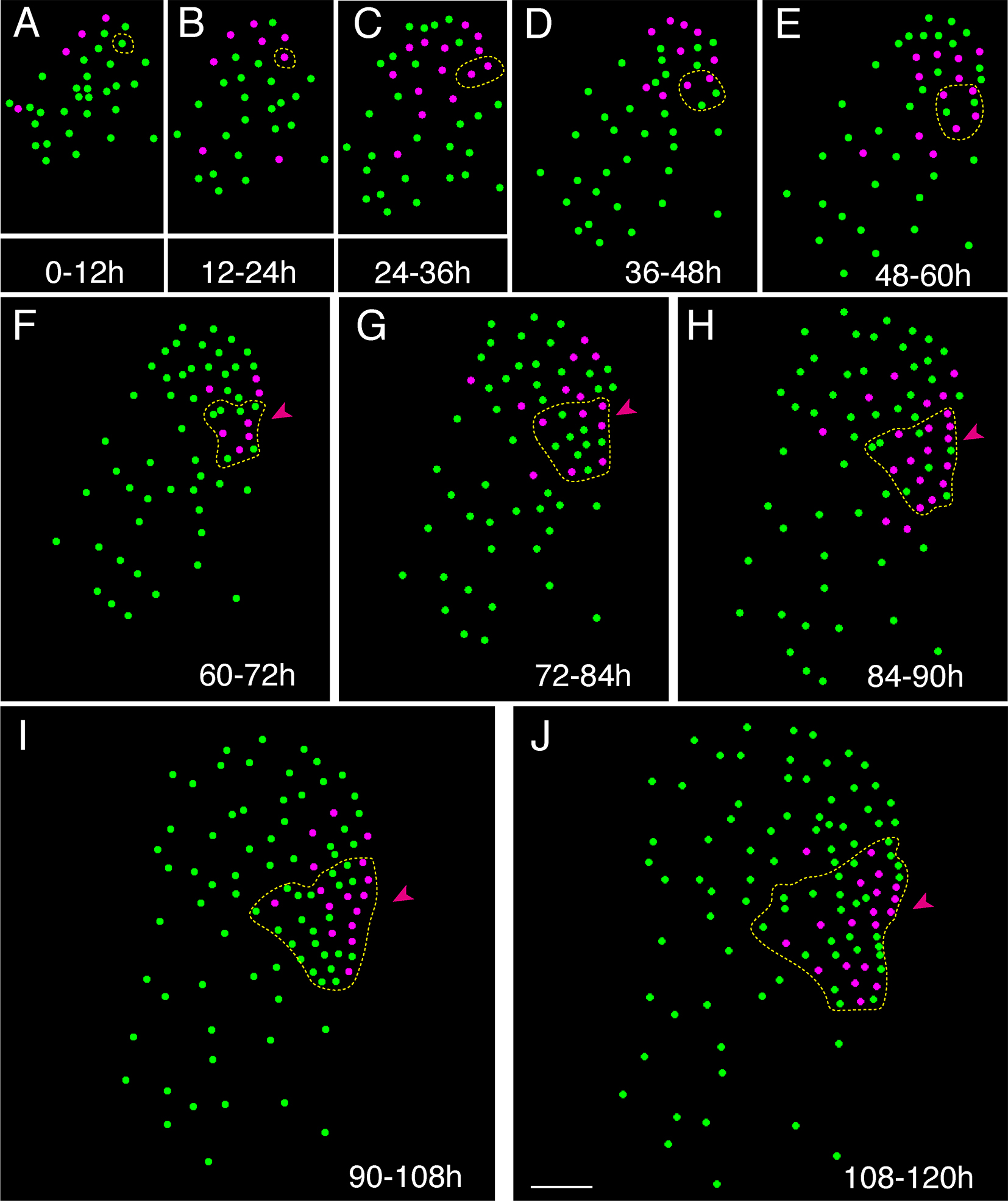

Supplement: S15 Fig — Each solid circle represents a single nucleus from the confocal images (S3 Fig). When an antheridium developed into a 3D complex structure, it was represented as a single solid circle for clear visualization, and subsequent division events within the antheridium were not included from the quantitative analysis. (A–J) Magenta solid circles indicate cells that underwent division, while green solid circles indicate cells that remained undivided during the indicated 12-h period. (A–J) Yellow dashed outlines highlight the MPC lineage over time. Magenta arrowheads (F–J) indicate the de novo formation of a multicellular meristem. Scale bar (J): 50 µm, applicable to all individual panels. Three independent samples were analyzed, showing comparable results. Cell division maps for the other two samples are included in S14 and S16 Figs, respectively. (JPG) [file pbio.3003592.s015.jpg]

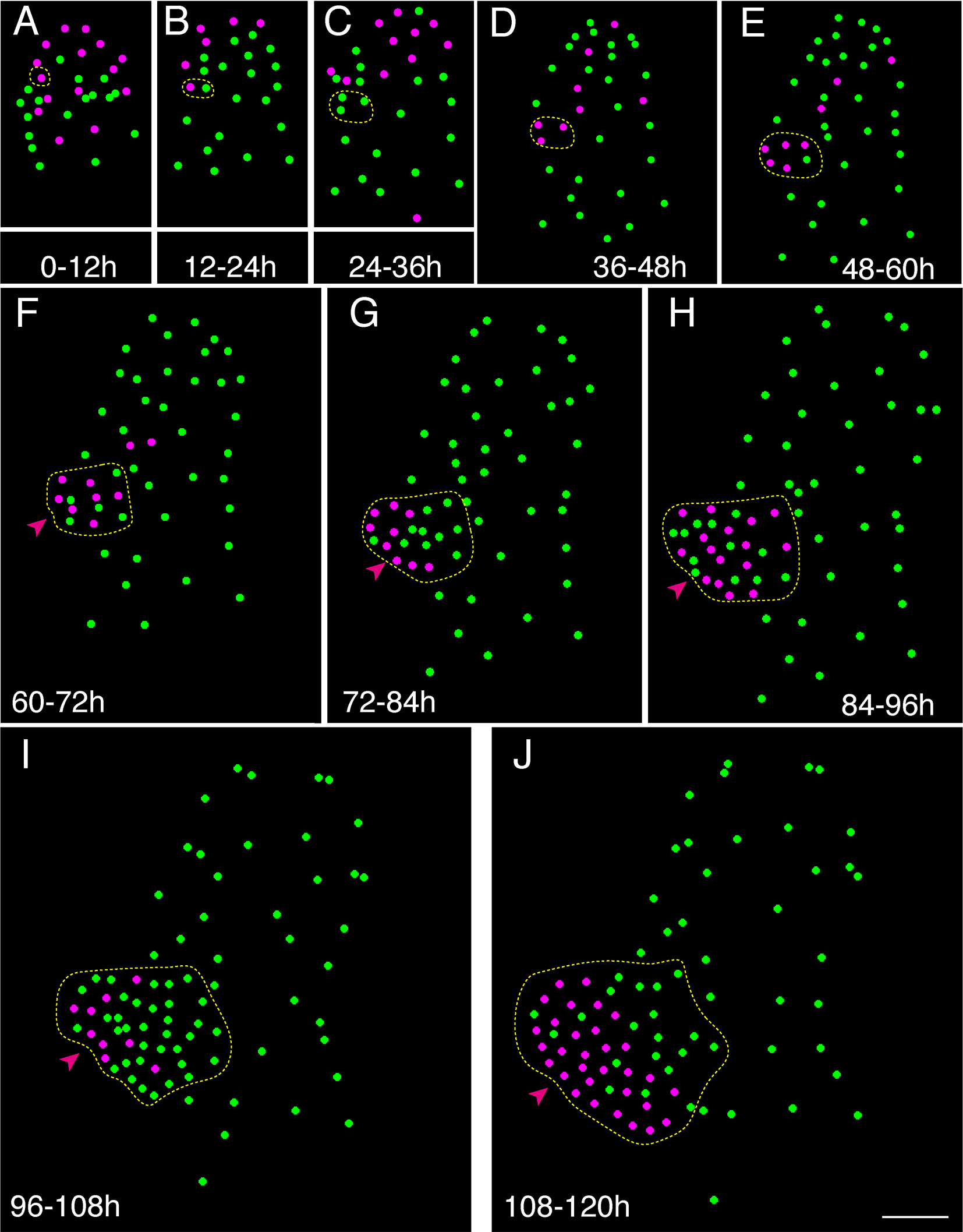

Supplement: S16 Fig — Each solid circle represents a single nucleus from the confocal images (S4 Fig). When an antheridium developed into a 3D complex structure, it was represented as a single solid circle for clear visualization, and subsequent division events within the antheridium were not included from the quantitative analysis. (A–J) Magenta solid circles indicate cells that underwent division, while green solid circles indicate cells that remained undivided during the indicated 12-h period. (A–J) Yellow dashed outlines highlight the MPC lineage over time. Magenta arrowheads (F–J) indicate the de novo formation of a multicellular meristem. Scale bar (J): 50 µm, applicable to all individual panels. Three independent samples were analyzed, showing comparable results. Cell division maps for the other two samples are included in S14 and S15 Figs, respectively. (JPG) [file pbio.3003592.s016.jpg]

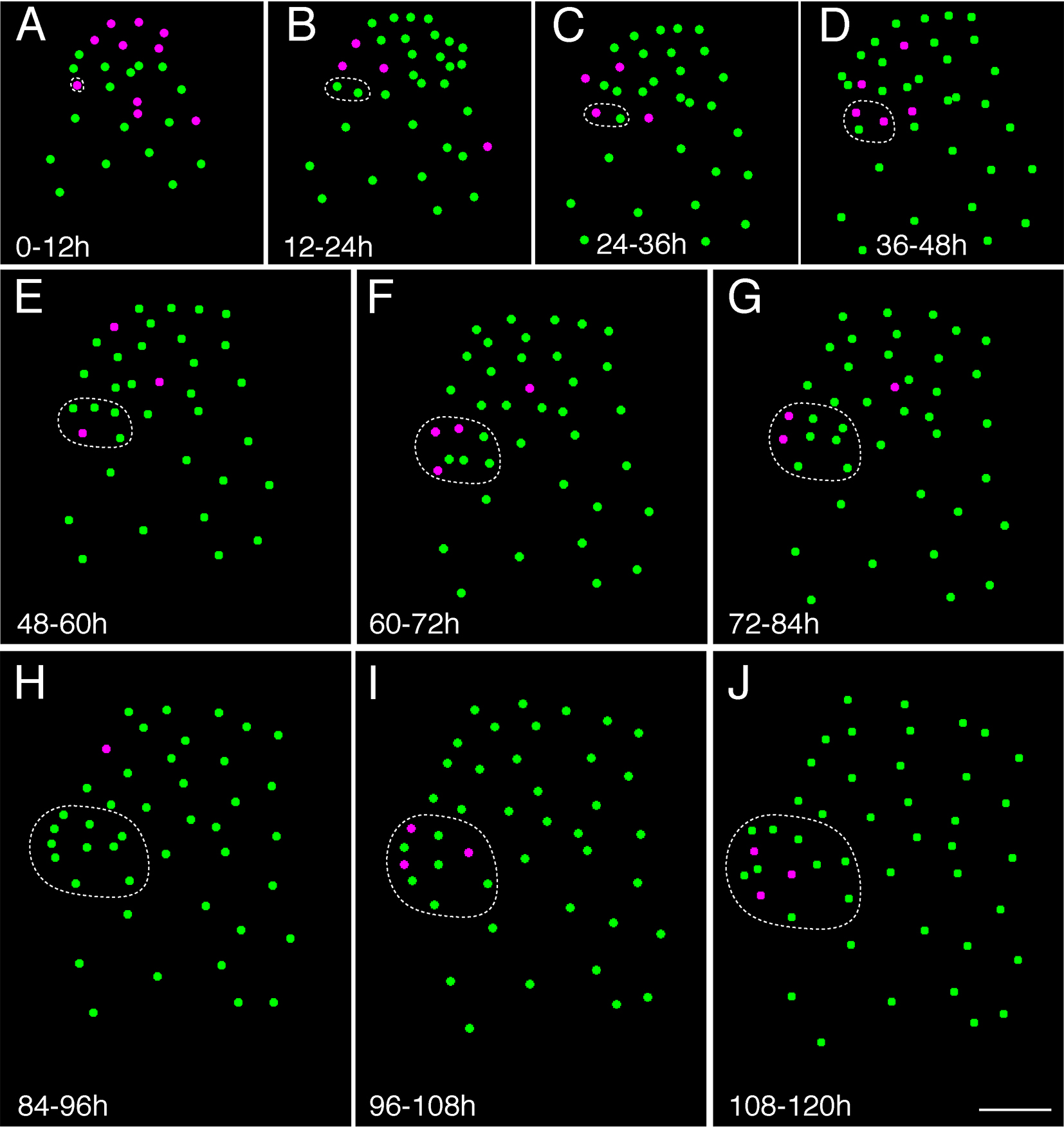

Supplement: S17 Fig — Each solid circle represents a single nucleus from the confocal images (S6 Fig). When an antheridium developed into a 3D complex structure, it was represented as a single solid circle for clear visualization, and subsequent division events within the antheridium were not included from the quantitative analysis. (A–J) Magenta solid circles indicate cells that underwent division, while green solid circles indicate cells that remained undivided during the indicated 12-h period. (A–J) White dashed outlines highlight the most actively dividing cell lineage over time. Scale bar (J): 50 µm, applicable to all individual panels. Three independent samples were analyzed, showing comparable results. Cell division maps for the other two samples are included in S14 and S18 Figs, respectively. (JPG) [file pbio.3003592.s017.jpg]

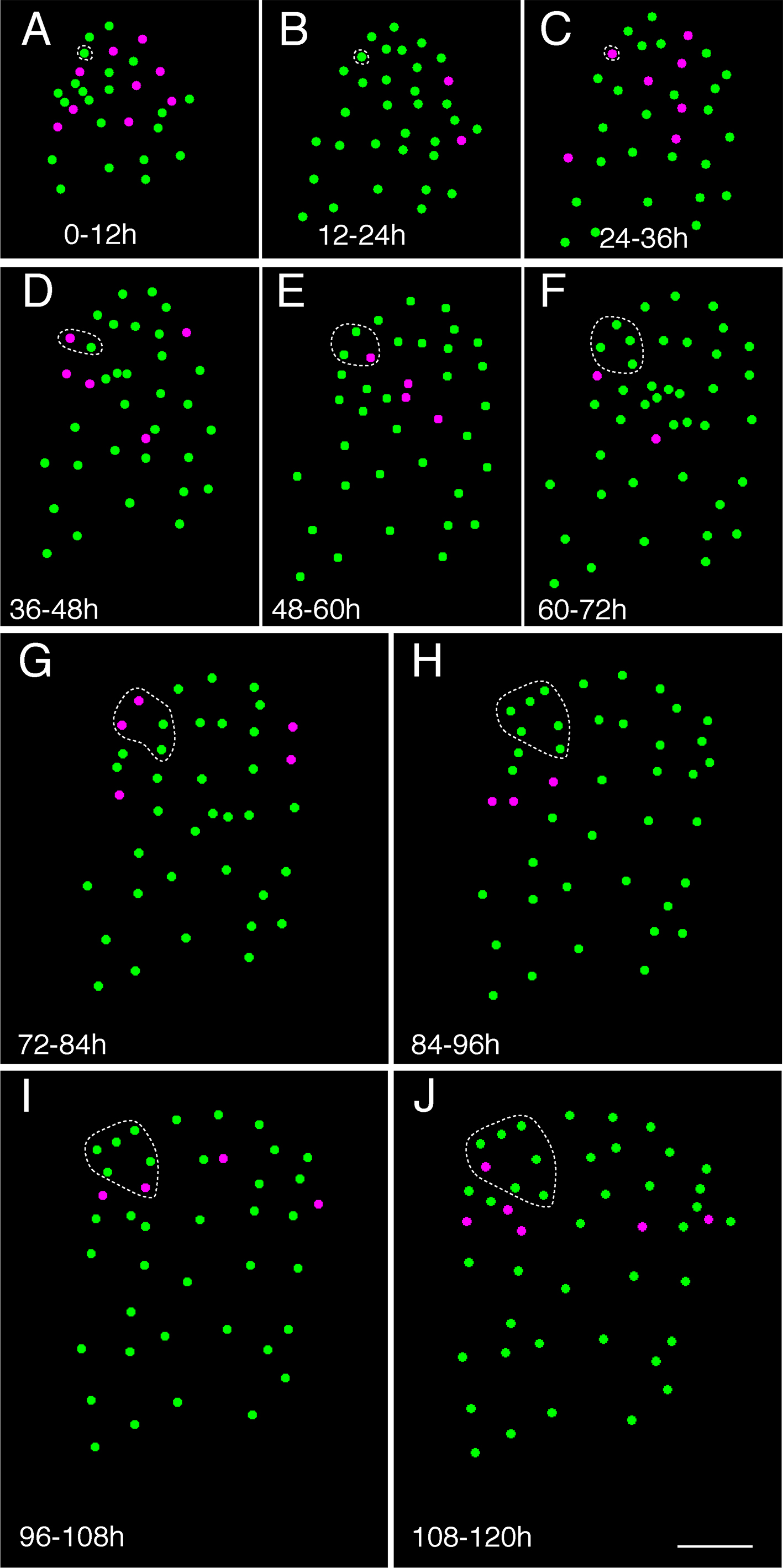

Supplement: S18 Fig — Each solid circle represents a single nucleus from the confocal images (S7 Fig). When an antheridium developed into a 3D complex structure, it was represented as a single solid circle for clear visualization, and subsequent division events within the antheridium were not included from the quantitative analysis. (A–J) Magenta solid circles indicate cells that underwent division, while green solid circles indicate cells that remained undivided during the indicated 12-h period. (A–J) White dashed outlines highlight the most actively dividing cell lineage over time. Scale bar (J): 50 µm, applicable to all individual panels. Three independent samples were analyzed, showing comparable results. Cell division maps for the other two samples are included in S14 and S17 Figs, respectively. (JPG) [file pbio.3003592.s018.jpg]

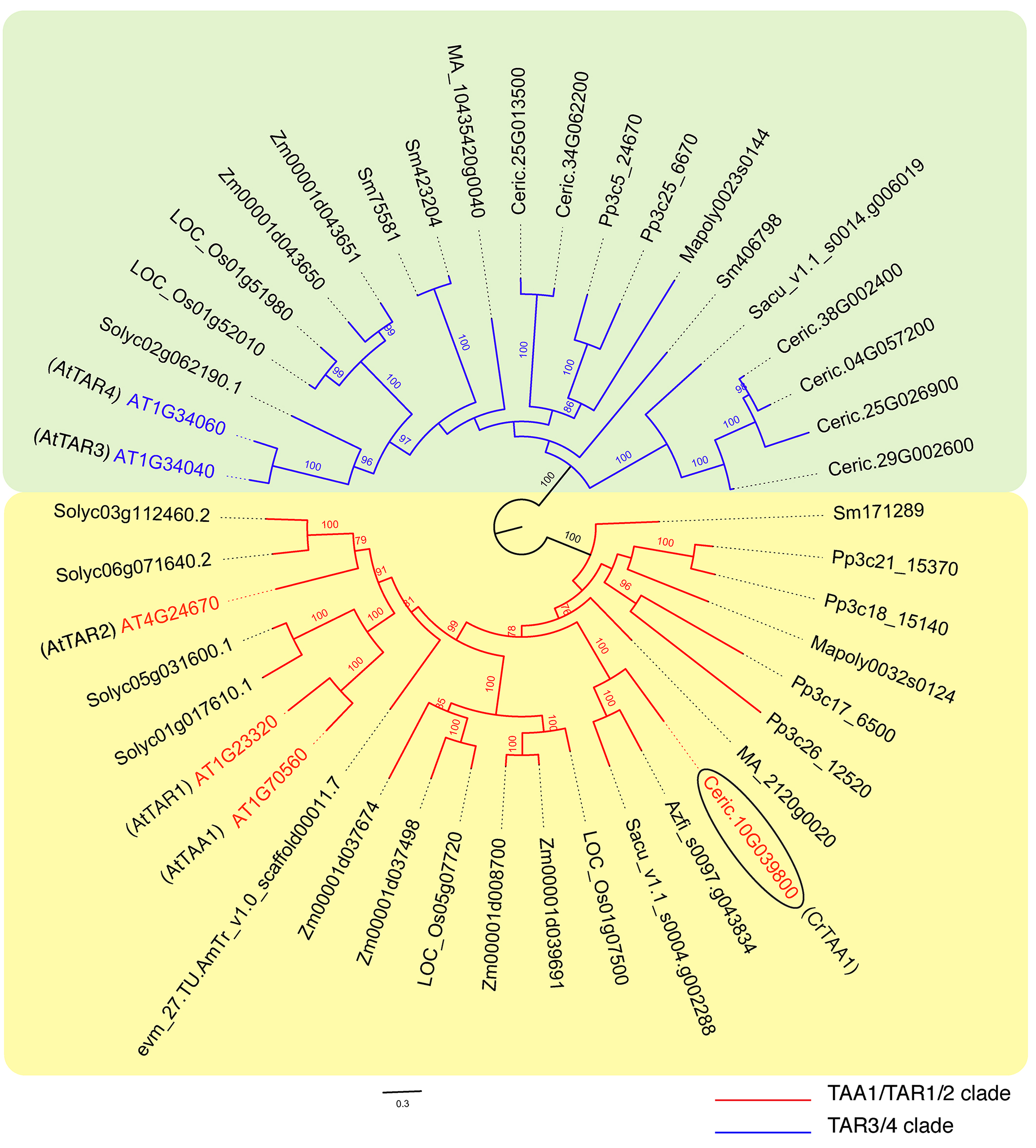

Supplement: S19 Fig — The maximum likelihood tree was constructed using IQ-TREE v2.4 with the JTT+I+R5 model and visualized using Figtree. The tree is midpoint-rooted. The TAA1/TAR1/2 clade is indicated by red lines, while the TAR3/4 clade is indicated by blue lines. Bootstrap values greater than 75 are shown, indicating well-supported branches. The sole TAA1 homolog in Ceratopteris (CrTAA1) is highlighted with a black oval. (JPG) [file pbio.3003592.s019.jpg]

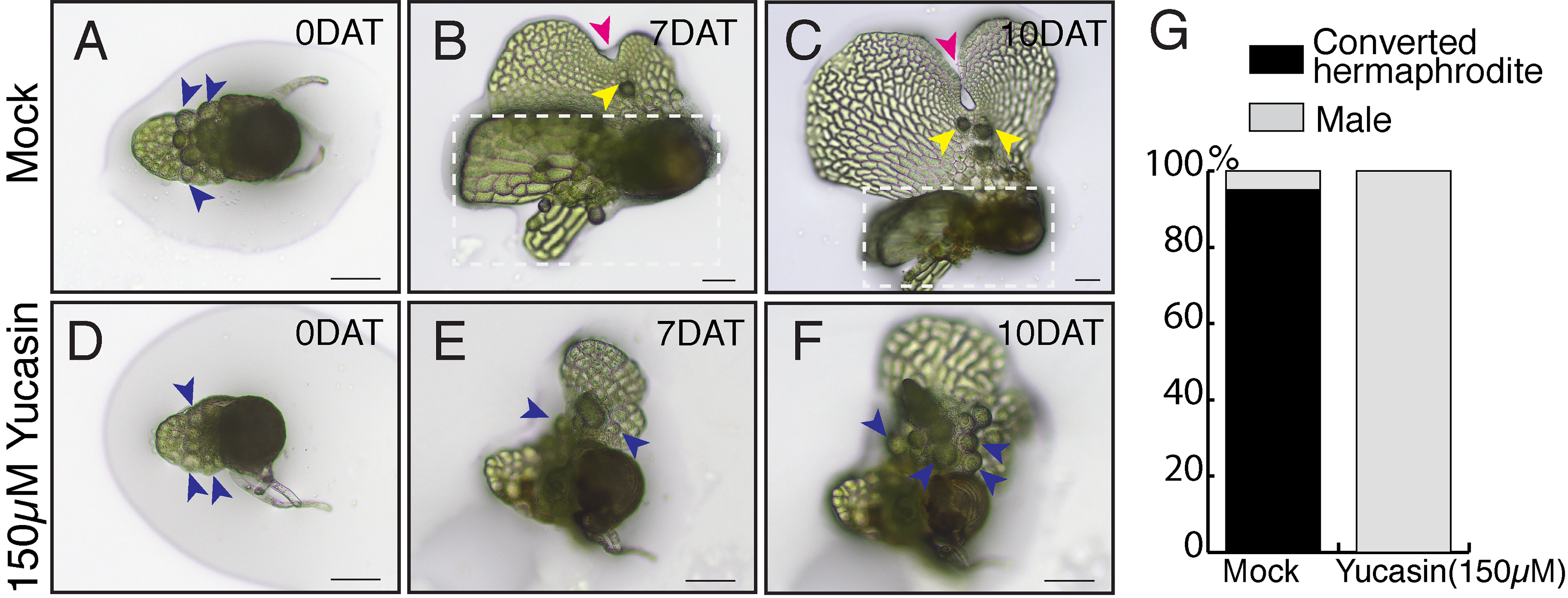

Supplement: S21 Fig — Light micrographs of representative male gametophytes at 0, 7, and 10 days after transfer (DAT) to antheridiogen-free FM, treated with either mock (A–C) or 150 µM Yucasin (D–F). In the mock-treated sample, white dashed rectangles in (B, C) indicate the original male body observed in (A). Magenta arrowheads (B, C) mark the newly formed meristem, yellow arrowheads (B, C) indicate newly formed archegonia, and blue arrowheads (A, D–F) highlight several antheridia. (G) The percentage of the successfully converted hermaphrodites was calculated from 20 independent gametophytes per treatment. Scale bars: 100 µm (A–F). The data underlying panel (G) can be found in S5 Data. (JPG) [file pbio.3003592.s021.jpg]
