# Supplementary material for: Dynamic auxin maxima regulate male-to-hermaphrodite conversion and de novo meristem formation in the fern Ceratopteris gametophytes
Source: PLoS Biol. 2026 Jan 23;24(1):e3003592. doi: 10.1371/journal.pbio.3003592 (PMC12829780; doi:10.1371/journal.pbio.3003592)
Supplement: S20 Fig — Protein sequences of CrTAA1 from the wild type and the mutant alleles crtaa1-1 (line 99) and crtaa1-2 (line 103) were aligned using Clustal Omega Multiple Sequence Alignment v1.2.4 (https://www.ebi.ac.uk/jdispatcher/msa/clustalo). Asterisks indicate the same amino acids, while dashes indicate missing or altered amino acids in the two CrTAA1 mutants. (PDF) [file pbio.3003592.s020.pdf]

```
Line 103    MTVESGCSICGTNTLPHPRKNFSKHEKRQLQMQNEDCTPYYDLYRKKRFKQYSCSPIQKL
Line 99     MTVESGCSICGTNTLPHPRKNFSKHEKRQLQMQNEDCTPYYDLYRKKRFKQYSCSPIQKL
WT          MTVESGCSICGTNTLPHPRKNFSKHEKRQLQMQNEDCTPYYDLYRKKRFKQYSCSPIQKL
            ************************************************************

Line 103    NYWLAASIFLNAICLSMLMGFIRLLAIKISRGPWRTEDVLT-------------------
Line 99     NYWLAASIFLNAICLSMLMGFIRPFWLSR-------------------------------
WT          NYWLAASIFLNAICLSMLMGFIRPSGYQDKQGALEDGGRSYVNGAGDSHLRYTNCSGQGR
            ***********************

Line 103    ------------------------------------------------------------
Line 99     ------------------------------------------------------------
WT          FEYDEINGGWCQCYSCFTGSICSEIIHDCVINFDHGDPKMFEKYWSLHQNTVSAVLRGNE

Line 103    ------------------------------------------------------------
Line 99     ------------------------------------------------------------
WT          RMSYFSDKEAVCWFLEPSLAMEIKLMHKMIGNAVTEGHHIVVGTGSSQLISAALYALSSL

Line 103    ------------------------------------------------------------
Line 99     ------------------------------------------------------------
WT          AHHRPVDVVSASPFYSSYPPMTNFLESQLHHWAGDASSYKTSGQNAYIELVTSPGNPDGM

Line 103    ------------------------------------------------------------
Line 99     ------------------------------------------------------------
WT          IHSAVVDGTGPVIYDLAYYWPHYTPITEAADYDIMLFTVSKTTGHAGTRIGWALVRDIEV

Line 103    ------------------------------------------------------------
Line 99     ------------------------------------------------------------
WT          AKKMTKYIELSTIGVSKDSQFRTAQILKGIRISYSDKAENSREYDSNSQRRLFHFGYEQM

Line 103    ------------------------------------------------------------
Line 99     ------------------------------------------------------------
WT          DLRWRQLRHAIGNSQCFSVVDFPSGYCQFFEKNTRAHPAFAWLYCEMDEDCHAVFKANGI

Line 103    -------------------------------------------
Line 99     -------------------------------------------
WT          LTRSGLHFGSSRKYIRISMLDHDNVFQLFIDRVHQMASSCHKK
```
