# Supplementary material for: Dynamic auxin maxima regulate male-to-hermaphrodite conversion and de novo meristem formation in the fern Ceratopteris gametophytes
Source: PLoS Biol. 2026 Jan 23;24(1):e3003592. doi: 10.1371/journal.pbio.3003592 (PMC12829780; doi:10.1371/journal.pbio.3003592)
Supplement: S22 Fig — (PDF) [file pbio.3003592.s022.pdf]

DNA sequences of the expression cassettes of gRNAs specific for *CrTAA1*.

Two gRNA sequences are underlined.

CTGCAGTCAAAGGTGTTGAGCCAAGTGACCAGCTAGGAGTCTCGTCAACACATGGGTGGG
GGGACTCTTGAGAGCCAAGTGGGGCATATTACTTTGATGATGGTTTCTTATTATTTGAGAGA
GTGGGTGCTTATTAGTTTCATGAGGAATGTTTGAGCCTTAGATCGTGGAGTGAGTGAATCT
CCATATATTCACGTCTCATTCCCTTTGCGCAAATATCAAAACATTTGATGATAGTAGAGTGA
AGGAAAGGATTTAAGAGGACCACGCGTCCGAAGTAAAAGACAAAAATAAAAAAGGGGCAC
ATGAATGCAACTTAATCTCAGTCGCTTGGTAATTTTAGTGCCCACAAAAGTTTGCGTCGCG
GGGTTTTTAAGGCCACAAAACCCACATATGCATGCGACCTTGATGGCAGCGGCCTACATAT
AAGGCTGCGCGCGCGTCCTTGCACTCG<u>ATGGGCTTCATCAGACCTTC</u>GTTTTAGAGCTAG
AAATAGCAAGTTAAAATAAGGCTAGTCCGTTATCAACTTGAAAAAGTGGCACCGAGTCGGT
GCTTTTTTTGTTTAAACAAGTTGTAATGAGTTGCTGGCCTCTCTTGGGATTGTTCATCAAAG
TAGCTGTCCCTACACACCACAACAGAATGGTGTTGTGGAGAGAAAGCACTGATATTAGGGT
TATAGGTGTATGTATATACGTGCGGAGCGGACAAAAAGAGCTCCTAAAGAGGGAAGAACG
TGAGGGGGTGGTGCTCAGTGTTGAACTTTCTATGAATGTAGTTTGTTGTAAAGATGCTTTCT
AGAATTGGAATTGGAGCTTTTTCTAGGATTCGAGGGTTTGTGGAATTGGAATTGGAGCTATT
TTTTAAGATATAATCTTGTTTCTGGGGCATTTGACTCCCATAGCTCCATATGTTTTGAGGTTC
TCGGTGCGTTTCCCTCTTCGTGATGGTGTTTGTCGTACTACCTTTGATCTCACTGTTTTGTT
CGTCCGTGTATTGCAGAGGGGTTTTGTGCCGATTGAAAAGCTGTGGCATTTTAGTGTGTTG
GTGTGTCGACTGAGGGTAGTTCAACGGAAGTGGCGCGCGTGGATAGGAATTATTAATAAT
GTGGGGAGGAGAAAACCCACATGTGAATCACAATCCTGAAAGATGAGCCTACATATTTCCC
GGCCAGCGATTGGCTGCTGTTG<u>ATATACTAACTGTTCGGGTC</u>GTTTTAGAGCTAGAAATAG
CAAGTTAAAATAAGGCTAGTCCGTTATCAACTTGAAAAAGTGGCACCGAGTCGGTGCTTTTT
TTGTTTAAACAAGTTGTAATGAGTTGCTGGCCTCTCTTGGGATTGTTCATCAAAGTAGCTGT
CCCTACACACCACAACAGAATGGTGTTGTGGAGAGAAAGCATAGCTGGGAACGCCTGCGC
GCGTCGCTGCTCATAATCCTCCTCGGCTGCTGCTAACAGACAGACAAATCTTCTTTCGAAA
TTGAAATTCTCTCTTTCGCACAGCCGTTTCATTAATGGCGGGCCTAAAATTGATAGGTAGGC
ATTTTGCCTGTCATTGGCCAGTTTTGAAACATCATGTCAAAATGCCAAGCTCTCGGATTGCT
AATTTGCCTTGTCAAATGAGAAGCTCCCTCGGAGCCATGGCTGCAG
